# PRMT1-mediated EZH2 methylation promotes breast cancer cell proliferation and tumorigenesis

**DOI:** 10.1038/s41419-021-04381-5

**Published:** 2021-11-13

**Authors:** Zhongwei Li, Diandian Wang, Xintian Chen, Wenwen Wang, Pengfei Wang, Pingfu Hou, Minle Li, Sufang Chu, Shuxi Qiao, Junnian Zheng, Jin Bai

**Affiliations:** 1 Cancer Institute, Xuzhou Medical University, Xuzhou, Jiangsu China; 2 Center of Clinical Oncology, Affiliated Hospital of Xuzhou Medical University, Xuzhou, Jiangsu China; 3 Intensive Care Unit, The Second Affiliated Hospital of Xuzhou Medical University, Xuzhou, Jiangsu China

**Keywords:** Breast cancer, Targeted therapies, Cell growth, Methylation, Phosphorylation

## Abstract

Protein arginine methyltransferase 1 (PRMT1) is able to promote breast cancer cell proliferation. However, the detailed mechanisms of PRMT1-mediated breast cancer cell proliferation are largely unknown. In this study, we reveal that PRMT1-mediated methylation of EZH2 at the R342 site (meR342-EZH2) has a great effect on PRMT1-induced cell proliferation. We also demonstrate that meR342-EZH2 can accelerate breast cancer cell proliferation in vitro and in vivo. Further, we show that meR342-EZH2 promotes cell cycle progression by repressing *P16* and *P21* transcription expression. In terms of mechanism, we illustrate that meR342-EZH2 facilitates EZH2 binding with SUZ12 and PRC2 assembly by preventing AMPKα1-mediated phosphorylation of pT311-EZH2, which results in suppression of *P16* and *P21* transcription by enhancing EZH2 expression and H3K27me3 enrichment at *P16* and *P21* promoters. Finally, we validate that the expression of PRMT1 and meR342-EZH2 is negatively correlated with pT311-EZH2 expression. Our findings suggest that meR342-EZH2 may become a novel therapeutic target for the treatment of breast cancer.

## Introduction

Protein arginine methyltransferase 1 (PRMT1), a member of the PRMT family, is able to catalyse many substrates (including histone and non-histone proteins), promoting arginine methylation [1]. It has been reported that PRMT1 is the major type I PRMT member, which can mediate the formation of monomethylarginine or asymmetric dimethylarginines (ADMAs) in its substrates [2]. Recently, many reports have demonstrated that the ectopic expression of PRMT1 in a variety of cancers plays a key role in cancer tumorigenesis and metastasis [3–10]. For instance, our previous study showed that overexpression of PRMT1 inhibits breast cancer cell senescence by increasing ZEB1 expression [11].

Enhancer of zeste homologue 2 (EZH2), SUZ12 and EED are the main components of PRC2 [12, 13]. EZH2 is a critical histone methyltransferase that is able to suppress PRC2 target gene transcription by mediating the trimethylation of Lys-27 in histone H3 (H3K27me3) [14, 15]. Many reports have demonstrated that EZH2 is ectopically expressed in cancer cells [16–18]. These studies also showed that high expression of EZH2 facilitates cancer cell proliferation and migration [16, 19]. A previous study found that SOX4 promotes breast cancer metastasis by increasing EZH2 transcription [17]. Our research also revealed that overexpression of EZH2 enhances breast cancer cell motility [20]. An increasing number of reports have demonstrated that a variety of EZH2 target genes are tumour suppressors, such as *HOXA7*, *HOXA9*, *DAB2IP*, *P16* and *P21* [18, 21–25].

As research progresses, an increasing number of studies have reported that post-translational modifications (PTMs) of EZH2 play key roles in EZH2 biological functions in cancer progression. PTMs of the EZH2 protein can regulate its stability, enzymatic activity, and PRC2 assembly [19]. For instance, AMPK-mediated EZH2-T311 phosphorylation attenuates PRC2 assembly by preventing the EZH2-SUZ12 interaction. It has also been shown that pT311-EZH2 inhibits ovarian cancer cell proliferation by suppressing EZH2-mediated H3K27me3 on PRC2 target genes [26]. A previous report clarified that AKT-mediated EZH2-S21 phosphorylation represses EZH2 methyltransferase activity, leading to a decrease in the amount of H3K27me3 on EZH2 target genes in breast cancer cells [27]. Our recent studies demonstrated that PRMT1-mediated EZH2-R342 ADMAs (meR342-EZH2) strengthen protein stability and enhance breast cancer metastasis [28, 29]. Interestingly, our previous study also showed that PRMT1-mediated meR342-EZH2 is positively correlated with tumour size in breast cancer patients [28]. Therefore, we wondered whether PRMT1-mediated EZH2 methylation is able to promote breast cancer cell proliferation and tumorigenesis.

Here we revealed that EZH2 is necessary for PRMT1 to enhance breast cancer cell proliferation. Moreover, PRMT1-mediated EZH2-R342 methylation can increase breast cancer cell proliferation and enhance breast cancer tumorigenesis. We also discovered that meR342-EZH2 is positively correlated with PRMT1 expression in breast cancer tissues, whereas meR342-EZH2 is negatively correlated with pT311-EZH2 expression in breast cancer tissues. Mechanistically, we disclosed that PRMT1-mediated meR342-EZH2 enhances PRC2 assembly and suppresses PRC2 target gene (i.e., *P16* and *P21*) transcription by preventing AMPK-mediated EZH2-T311 phosphorylation. Our study showed that targeting meR342-EZH2 may become a therapeutic strategy for the treatment of breast cancer.

## Materials and methods

### Cell lines and cell culture

MDA-MB-231 cells were cultured in L-15 medium with 10% fetal bovine serum (FBS) at 37 °C without CO_2_. MCF7 cells were cultured in RPMI-1640 medium with 10% FBS. HEK293T cells were cultured in Dulbecco’s modified Eagle medium with 10% FBS. All the cell lines were obtained from the American Type Culture Collection.

### Reagents and plasmids

Reagents (including chemicals, medium, FBS, etc.) and sequences of short hairpin RNA used in this study are shown in the Supplementary Information. The construction of shEZH2-3′UTR plasmid, Flag-EZH2-WT and Flag-EZH2-R342K expression plasmids are described in our previous study [28].

### Stable cell line generation

Lentiviruses were produced by co-transfecting HEK293T cells with the expression plasmids and the packaging plasmids (psPAX2 and pMD2.G). The supernatants were collected after 48 h and filtered through 0.45 μm filters (Millipore, Temecula, CA, USA), then concentrated using Amicon Ultra centrifugal filters (Millipore 100KD MWCO). The concentrated viruses were used to infect MCF7 and MDA-MB-231 cells. Stable transfection cell lines were selected with 2 mg/ml puromycin for 15 days. Stable overexpression or knockdown of PRMT1 and EZH2 cells were generated by lentivirus infection.

### Cell proliferation and colony formation assays

Cell Counting Kit-8 (CCK-8) assay was used to detect cell proliferation ability according to the CCK-8 manufacturer’s protocol (Dojindo). For colony formation assay, 1 × 10^3^ cells were cultured in 60 mm plate at 37 °C for 14 days. Visible colonies were washed twice with phosphate-buffered saline (PBS), fixed and stained with 4% paraformaldehyde and crystal violet, respectively. The number of colonies was counted visually.

### Cell cycle analysis

Cells cycle analysis was performed as previously described [30]. The detailed procedure was described in the Supplementary Information.

### RNA extraction, reverse transcription and qRT-PCR

These relevant protocols were carried out as previously described [20]. The cDNA Synthesis SuperMix and Top Green qPCR SuperMix were purchased from TransGen Biotech Company (AT311-03; AQ131-03). The sequences of real-time quantitative reverse transcription PCR (qRT-PCR) primers are listed in Supplementary Information.

### Western blotting, IP and Co-IP assays

Western blotting, immunoprecipitation (IP) and coimmunoprecipitation (Co-IP) were performed as described previously [29]. Specific primary antibodies against GAPDH (60004-1-AP, Proteintech), PRMT1 (11279-1-AP, Proteintech), Flag-Tag (KM8002, SUNGENE BIOTECH), EZH2 (3147; 5246, CST), CDK4 (12790, CST), AMPKα1(2795, CST), EED (16818-1-AP, Proteintech), SUZ12 (20366-1-AP, Proteintech), pT311-EZH2 (27888S, CST), P16 (A0262, ABclonal), P21 (10355-1-AP, Proteintech), Cyclin B1 (28603-1-AP, Proteintech), and Cyclin E2 (4132T, CST) were used for western blot assays.

### Chromatin immunoprecipitation

Chromatin immunoprecipitation (ChIP) assay was performed using the Simple ChIP Enzymatic Chromatin IP Kit (CST, Cat#9004) according to the manufacturer’s protocol. The ChIP primers for P16 and P21 promoter sequences are shown in Supplementary Information.

### Antibody generation and detection

The anti-ADMA-R342-EZH2 (anti-meR342-EZH2) antibody was generated as described previously [28].

### Breast cancer tissues and immunohistochemistry assay

The breast cancer tissue microarrays (TMAs) for staining PRMT1, meR342-EZH2 and pT311-EZH2 were purchased from Avilabio Company and contain 75 cases of breast cancer tissue specimens. Immunohistochemistry (IHC) assays were carried out using a standard streptavidin-Peroxidase method in our previous report [31]. Heat-induced epitope retrieval was performed with retrieval buffer (EDTA pH 9.0 or citrate buffer pH 6.0) before the IHC staining protocol. For primary antibody, the anti-PRMT1 antibody was used with 1:200 dilution, the anti-meR342-EZH2 was used with 1:50 dilution and the anti-pT311-EZH2 antibody was used with 1:100 dilution. The slide without primary antibody incubation served as the negative control. The detailed method of IHC assessment was described in the Supplementary Information.

### Animal work of tumour xenograft model

The animal experiments were approved by the Animal Care Committee of Xuzhou Medical University, Xuzhou, China. Female BALB/c nude mice (6–8 weeks old) were obtained from the Beijing Vital River Laboratory Animal Technology Co., Ltd. MDA-MB-231-Vector, MDA-MB-231-EZH2-WT and MDA-MB-231-EZH2-R342K cells (5 × 10^6^ cells) were injected subcutaneously into the nude mice. Four weeks later, the mice were killed and the tumours were weighed and detected the expression of EZH2, meR342-EZH2, P16, P21 and Ki67 by IHC. The following is the brief protocol of animal treatment experiment by GSK3368715 (short for GSK715; MCE, Cat#HY-128717A). MDA-MB-231 cells (5 × 10^6^ cells) were injected subcutaneously into the nude mice. Three weeks later, GSK715 (100 mg/kg, each day) or PBS were treated with nude mice by intraperitoneal injection. After 10 days treatment with GSK715 or PBS, the nude mice were killed and the tumours were weighed and detected the expression of meR342-EZH2 and Ki67 by IHC.

### Statistical analysis

For the TMA slides, statistical analysis was performed with SPSS 20 software (SPSS, Inc., Chicago, IL). The Student’s *t*-test was used to determine statistical significance of differences between groups. *p* < 0.05 was considered statistically significant. Data are presented as mean ± SEM. Statistical analysis was performed using the GraphPad Prism software (GraphPad Software, La Jolla, CA, USA).

## Results

### PRMT1 promotes cell proliferation by mediating meR342-EZH2 in breast cancer

In our previous study, we found that PRMT1 catalyses EZH2-R342 ADMA generation. PRMT1-mediated meR342-EZH2 is able to enhance breast cancer cell migration and metastasis in vitro and in vivo. Interestingly, our data also showed that meR342-EZH2 is positively correlated with tumour diameter in clinicopathological indicators in patients with breast cancer. This suggests that PRMT1-mediated meR342-EZH2 may promote breast cancer cell proliferation and tumorigenesis in vitro and in vivo.

**Fig. 1:**
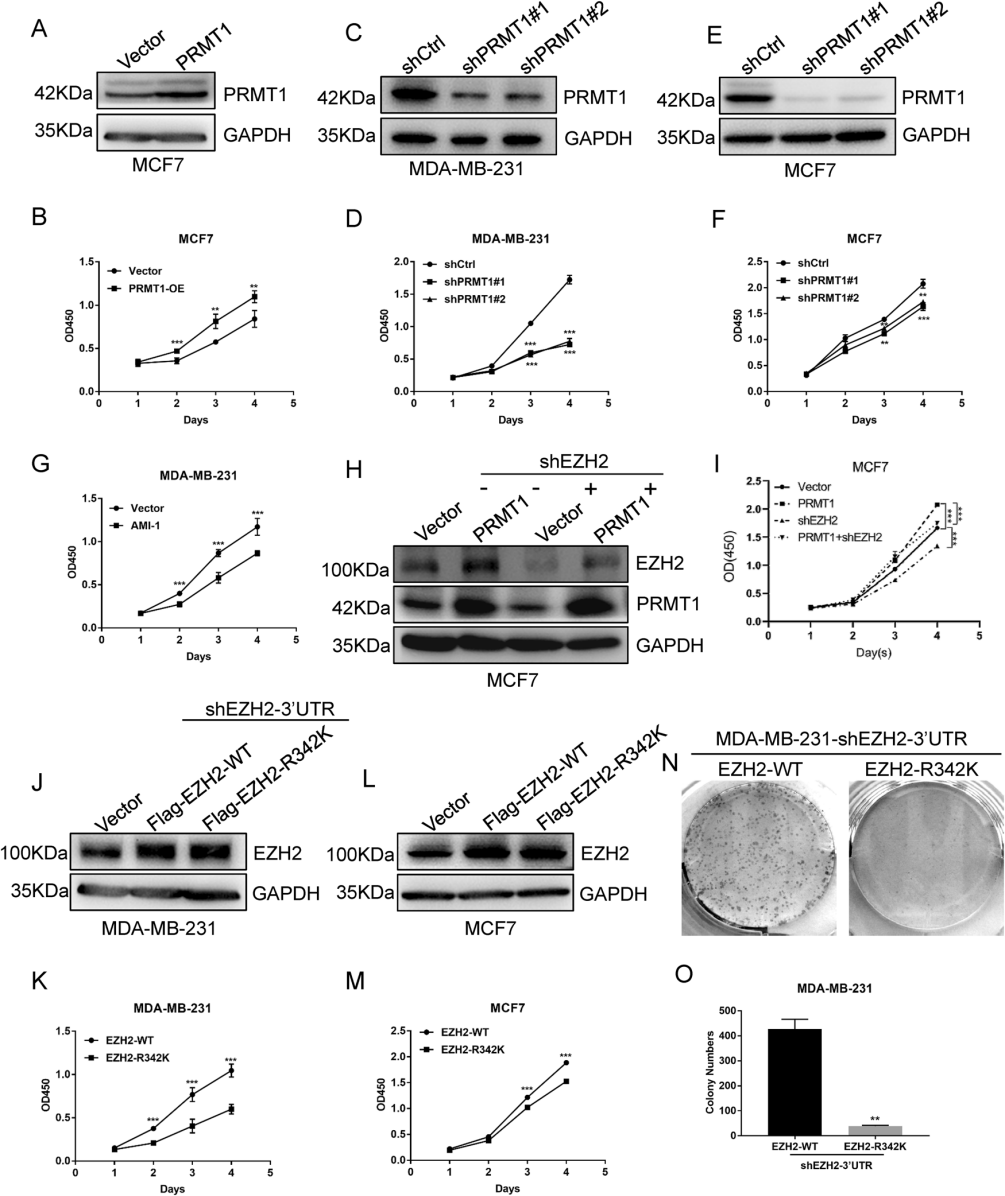
PRMT1-mediated meR342-EZH2 facilitates cell proliferation in breast cancer. **A** Western blotting detected PRMT1 expression in MCF7-Vector and MCF7-PRMT1 cells. **B** CCK-8 assay measured cell proliferation ability of MCF7-Vector cells and MCF7-PRMT1 cells. **C**–**F** Western blotting confirmed PRMT1 knockdown in MCF7 and MDA-MB-231 cells (**C** and **E**); detection of the effect of knockdown PRMT1 on cell proliferation in MCF7 and MDA-MB-231 by CCK-8 assays (**D** and **F**). **G** CCK-8 detected the MCF7 cell proliferation ability after treated with PRMT1-specific inhibitor AMI-1 (0.8 μM). **H**, **I** Western blotting detected PRMT1 and EZH2 expression in MCF7-Vector, MCF7-PRMT1, MCF7-shEZH2 and MCF7-(PRMT1 + shEZH2) (**H**); CCK-8 analysed the cell proliferation ability in these cell lines (**I**). **J**–**M** CCK-8 detected overexpression of EZH2-WT or EZH2-R342K mutant impact on cell proliferation of MCF7 and MDA-MB-231 cells. **N**, **O** Detection cell proliferation ability of MDA-MB-231-EZH2-WT and MDA-MB-231-EZH2-R342K by clone formation assays. All the above experiments were replicated three times for statistical analyses (*n* = 3). Data are represented as mean ± SEM and *p < 0.05, **p < 0.01, ***p < 0.001 (Student’s *t*-test).

To test this hypothesis, we first showed that overexpression of PRMT1 promoted cell proliferation in MCF7 breast cancer cells by CCK-8 assays (Fig. 1A, B), whereas knockdown of PRMT1 inhibited cell proliferation in MCF7 and MDA-MB-231 breast cancer cells (Fig. 1C–F). In addition, the cell proliferation ability was attenuated when MDA-MB-231 cells were treated with the PRMT1-specific inhibitor AMI-1 (Fig. 1G). In addition, we also demonstrated that GSK715, as a promising clinical-trialled type I PRMTs inhibitor, can repress breast cancer cell proliferation (Supplementary Fig. S1). These data suggest that PRMT1 can facilitate breast cancer cell proliferation.

We then knocked down EZH2 in MCF7-PRMT1 cells to detect breast cancer cell proliferation. Our results showed that the cell proliferation ability of MCF7-PRMT1 cells was dramatically decreased when EZH2 expression was silenced (Fig. 1H, I). This suggested that EZH2 is required for PRMT1 to facilitate breast cancer cell proliferation. Moreover, we want to explore whether EZH2-R342 ADMA modification is necessary for breast cancer cell proliferation through ectopic expression EZH2-WT or EZH2-R342K in MCF7 and MDA-MB-231 cells. As our previous study have showed, the expression level of EZH2 in MDA-MB-231 cells is high [28]. To eliminate the influence of high background EZH2 expression in this cell line, we first decreased endogenous EZH2 expression using lentivirus targeting the 3′-untranslated region (UTR) of EZH2 mRNA in MDA-MB-231 cells (MDA-MB-231-shEZH2-3′UTR). Then, we transfected lentiviruses expressing Flag-EZH2-WT or Flag-EZH2-R342K into MDA-MB-231-shEZH2-3′UTR cells. We confirmed the ectopic expression level of Flag-EZH2 proteins is equally in MDA-MB-231-shEZH2-3′UTR-Flag-EZH2-WT cells and MDA-MB-231-shEZH2-3′UTR-Flag-EZH2-R342K cells by western blot assays (Fig. 1J).

Interestingly, our CCK-8 assay results showed that ectopic expression of Flag-EZH2-WT dramatically facilitates breast cancer cell proliferation. At the same time, overexpression of Flag-EZH2-R342K had little effect on the proliferation of MCF7 and MDA-MB-231 breast cancer cells (Fig. 1J–M). Consistently, the colony formation assays also demonstrated that only ectopic expression of wild-type EZH2 enhanced MDA-MB-231 cell colony formation ability compared with the overexpression of R342K mutant EZH2 (Fig. 1N, O). In summary, our results indicate that PRMT1-mediated meR342-EZH2 can enhance cell proliferation in breast cancer cells.

### PRMT1-mediated meR342-EZH2 accelerates the cell cycle of breast cancer cells

**Fig. 2:**
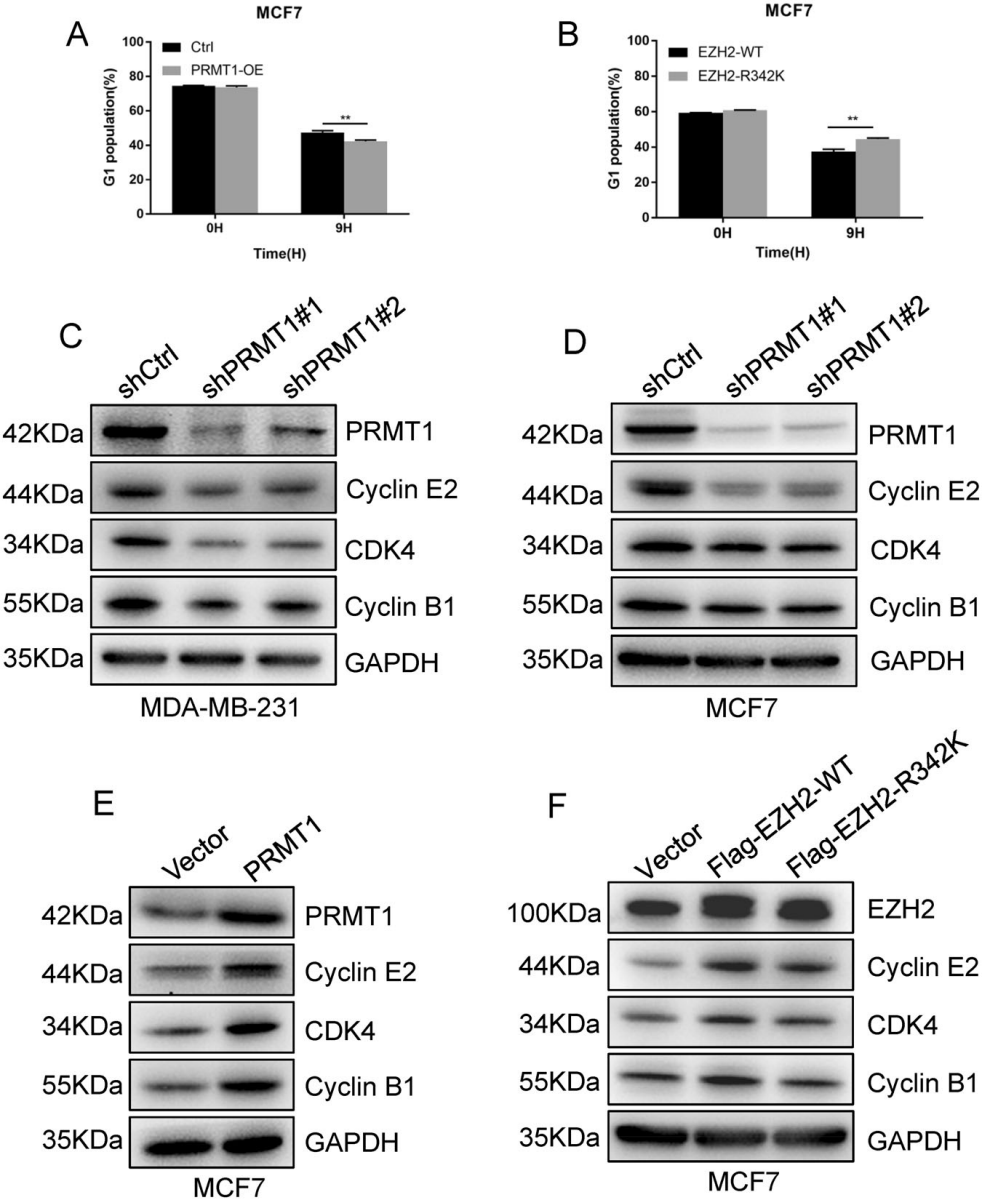
PRMT1-mediated meR342-EZH2 promotes breast cancer cell cycle. **A** Detection of G1 phase of cell cycle in MCF7-Ctrl and MCF7-PRMT1 cells. **B** Detection of G1 phase of cell cycle in MCF7-EZH2-WT and MCF7-EZH2-R342K cells. **C**, **D** Western blotting measured Cyclin E2, CDK4, Cyclin B1 and PRMT1 expression when knocked down PRMT1 in MCF7 and MDA-MB-231 cells. **E**, **F** Immunoblot analysed Cyclin E2, CDK4, Cyclin B1 and PRMT1 expression in MCF7-Vector and MCF7-PRMT1 cells (**E**), or MCF7-Vector, MCF7-EZH2-WT and MCF7-EZH2-R342K cells (**F**). All the above experiments were replicated three times for statistical analyses (*n* = 3). Data are represented as mean ± SEM and **p < 0.01 (Student’s *t*-test).

To explore potential mechanisms for this regulation, we next sought to examine whether meR342-EZH2 can affect the cell cycle progression of breast cancer cells. To this end, we performed cell cycle analysis in MCF7-Ctrl and MCF7-PRMT1 cells, and MCF7-Flag-EZH2-WT and MCF7-Flag-EZH2-R342K cells. We found that ectopic expression of PRMT1 decreased the percentage of cells in G0/G1 phase (Fig. 2A). Meanwhile, the percentage of cells in G0/G1 phase was upregulated in R342K mutant EZH2 cells compared with wild-type EZH2 cells (Fig. 2B). This suggested that PRMT1-mediated meR342-EZH2 may promote cell cycle progression.

We then detected the expression of several key cell cycle-related proteins in these cells. On the one hand, we found that knockdown of PRMT1 decreased Cyclin E2, Cyclin B1 and CDK4 expression in MCF7 and MDA-MB-231 breast cancer cells (Fig. 2C, D); on the other hand, we also confirmed that ectopic expression of PRMT1 increased the expression of these mentioned proteins (Fig. 2E). Moreover, we revealed that ectopic expression of Flag-EZH2-WT led to strongly increased expression of these cell cycle-related proteins in MCF7 cells. At the same time, overexpression of Flag-EZH2-R342K had an insignificant effect on the expression of these proteins in MCF7 cells (Fig. 2F). Taken together, our results suggest that PRMT1-mediated meR342-EZH2 plays a critical role in the cell cycle progression of breast cancer. PRMT1 facilitating cell cycle progression in breast cancer is likely dependent on PRMT1-mediated meR342-EZH2.

### PRMT1-mediated meR342-EZH2 strengthens PRC2 assembly by inhibiting AMPK-mediated EZH2-T311 phosphorylation

**Fig. 3:**
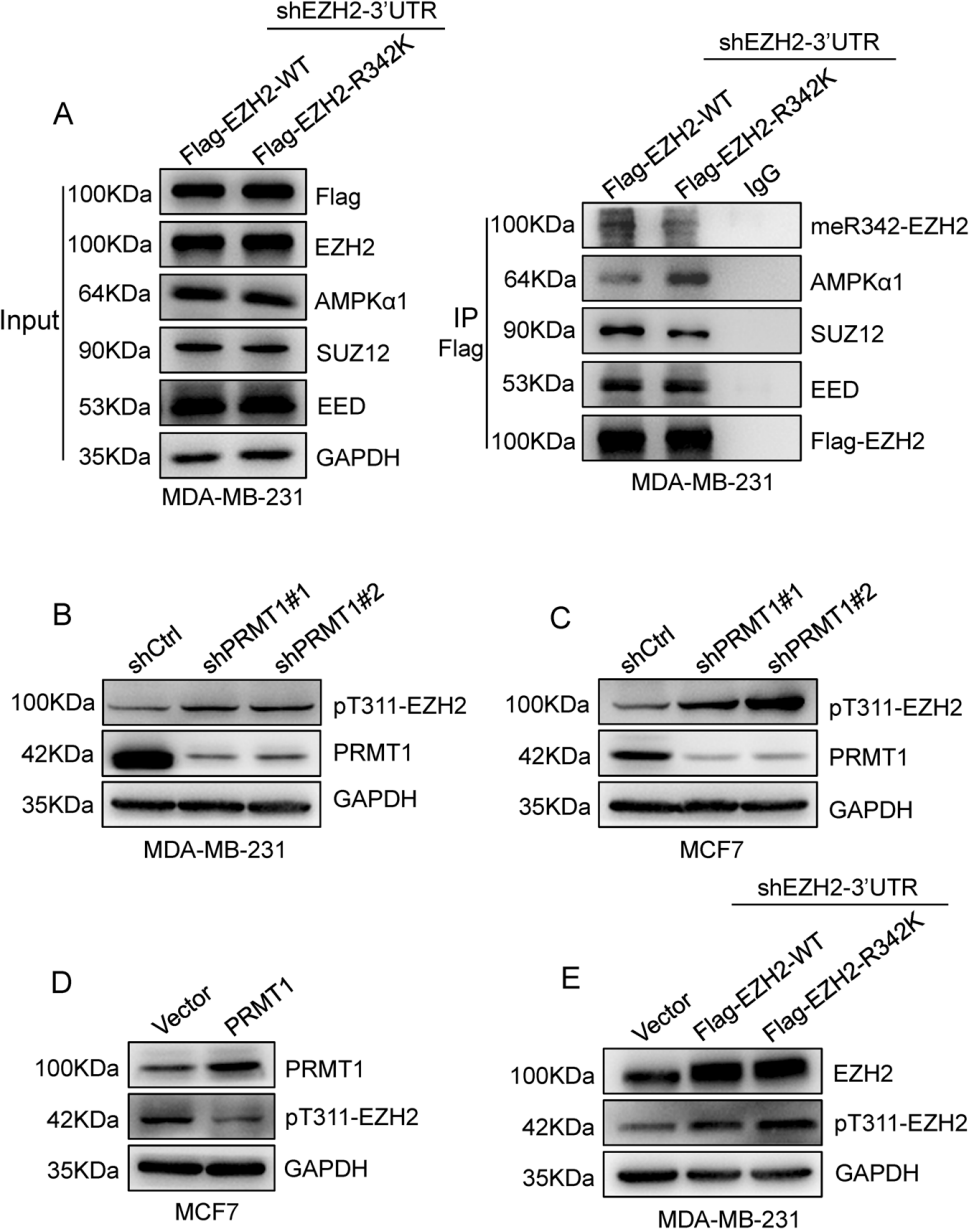
PRMT1-mediated meR342-EZH2 enhanced PRC2 assembly through inhibiting AMPK-mediated pT311-EZH2. **A** Western blotting detecting EZH2 binding with meR342-EZH2, AMPKα1, SUZ12, EED after Co-IP Flag-EZH2 in MCF7-Flag-EZH2-WT and MCF7-Flag-EZH2-R342K cells. **B**, **C** Detection of pT311-EZH2 expression after knockdown PRMT1 in MDA-MB-231 (**B**) and MCF7 (**C**) cells. **D**, **E** Immunoblot analysed pT311-EZH2 expression in MCF7-Vector and MCF7-PRMT1 cells (**D**), or MDA-MB-231-Vector, MDA-MB-231-EZH2-WT and MDA-MB-231-EZH2-R342K cells (**E**).

Next, we wanted to determine the mechanism by which PRMT1-mediated meR342-EZH2 promotes cell cycle progression. First, we found that the amount of EZH2-R342K binding with SUZ12 was strongly decreased in Flag-EZH2-R342K MDA-MB-231 cells ectopically expressing Flag-EZH2-R342K compared with Flag-EZH2-WT MDA-MB-231 cells, as determined by Co-IP assays. In contrast, the amount of EZH2-EED association was not so much changed between the EZH2-WT group and the EZH2-R342K group in MDA-MB-231 cells (Fig. 3A). In addition, we confirmed that the ability of EZH2 to interact with SUZ12 was decreased in the EZH2-R342K group compared with the EZH2-WT group in HEK293T-Flag-EZH2-WT and HEK293T-Flag-EZH2-R342K cells (Supplementary Fig. S2). Therefore, we speculated that meR342-EZH2 might strengthen PRC2 assembly by enhancing the EZH2-SUZ12 interaction.

Recently, Wei and colleagues [26] reported that AMPKα1-mediated EZH2-T311 phosphorylation prevents EZH2-SUZ12 association and PRC2 assembly. Our Co-IP-Flag-EZH2 experiments confirmed that the EZH2-R342K group strongly enhanced EZH2 binding with AMPKα1 compared with that in the EZH2-WT group in MDA-MB-231 cells and HEK293T cells (Fig. 3A). Moreover, our Co-IP assays obtained similar results in HEK293T cells ectopically expressing Flag-EZH2-WT or Flag-EZH2-R342K (Supplementary Fig. S2). Several studies have confirmed that protein arginine methylation is able to crosstalk with protein phosphorylation. Combined with our data, we proposed that PRMT1-mediated meR342-EZH2 may inhibit AMPKα1-mediated pT311-EZH2.

To test our hypothesis, we detected pT311-EZH2 expression by the anti-pT311-EZH2-specific antibody in expressing shCtrl or shPRMT1 breast cancer cells. The data showed that pT311-EZH2 expression was increased when PRMT1 was knocked down in both MCF7 and MDA-MB-231 cells (Fig. 3B, C). The amount of pT311-EZH2 was decreased when PRMT1 was overexpressed in MCF7 cells (Fig. 3D). Moreover, we also demonstrated that R342K mutant EZH2 strongly increased pT311-EZH2 expression compared with wild-type EZH2 in MDA-MB-231-shEZH2-3′UTR cells (Fig. 3E). Taken together, our data suggest that PRMT1-mediated meR342-EZH2 inhibits SUZ12 binding with EZH2 by preventing AMPKα1-mediated pT311-EZH2 in breast cancer.

### PRMT1-mediated meR342-EZH2 promotes cell proliferation by suppressing *P16* and *P21* transcriptional expression in breast cancer

**Fig. 4:**
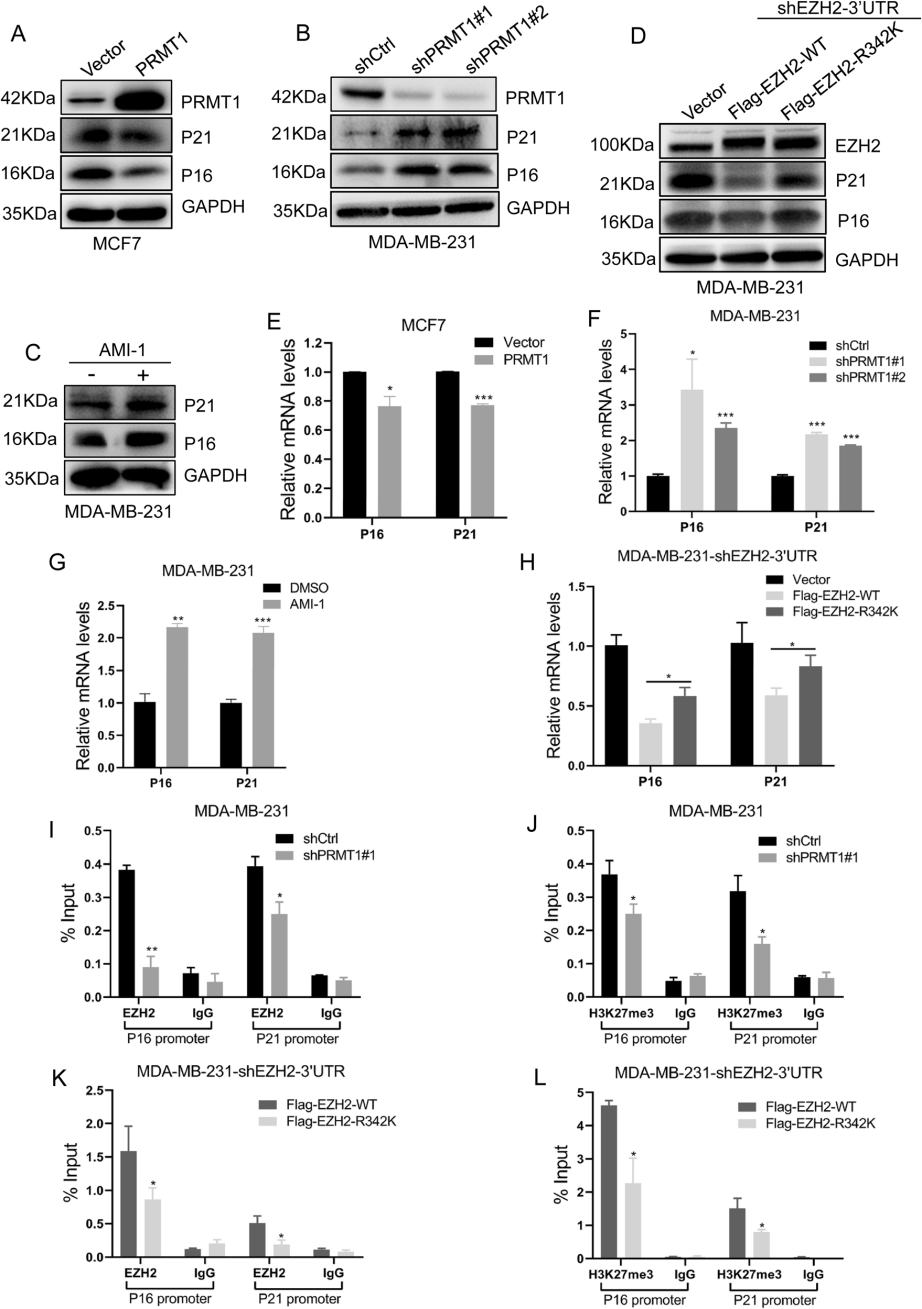
PRMT1-mediated meR342-EZH2 strengthens suppression of *P16* and *P21* transcriptional expression. **A**–**D** Western blotting detection of P16 and P21 expression after PRMT1 overexpression (**A**) or knockdown PRMT1 (**B**), or treated with PRMT1 (**C**) inhibitor AMI-1 (0.8 μM) or ectopic expression of Flag-EZH2-WT and Flag-EZH2-R342K (**D**) in MCF7 and MDA-MB-231 cells, respectively. **E**–**H** qRT-PCR detection of P16 and P21 expression after PRMT1 overexpression (**E**) or knockdown PRMT1 (**F**), or treated with PRMT1 (**G**) inhibitor AMI-1 (0.8 μM) or ectopic expression of Flag-EZH2-WT and Flag-EZH2-R342K (**H**) in MCF7 and MDA-MB-231 cells, respectively. **I**, **J** ChIP assays analysed EZH2 (**I**) and H3K27me3 (**J**) enrichment on P16 and P21 promoters in MDA-MB-231-shCtrl and MDA-MB-231-shPRMT1 cells. **K**, **L** Detection the enrichment of EZH2 (**K**) and H3K27me3 (**L**) on *P16* and *P21* promoters in MDA-MB-231-EZH2-WT and MDA-MB-231-EZH2-R342K cells by ChIP experiments. All the above experiments were replicated three times for statistical analyses (*n* = 3). Data are represented as mean ± SEM of three independent experiments and *p < 0.05, **p < 0.01, ***p < 0.001 (Student’s *t*-test).

Furthermore, we tried to detect the expression of several major proliferation-associated proteins. We showed that overexpression of PRMT1 abrogated P16 and P21 expression at the protein and transcriptional levels (Fig. 4A, E), whereas knockdown of PRMT1 increased P16 and P21 expression at both the protein and mRNA levels in MCF7 and MDA-MB-231 cells (Fig. 4B, F and Supplementary Fig. S3). We also found that the PRMT1 inhibitor AMI-1 increased P16 and P21 protein and mRNA expression levels (Fig. 4C, G). Subsequently, we revealed that only overexpression of EZH2-WT could disrupt P16 and P21 expression compared with ectopic expression of mutant EZH2-R342K in MDA-MB-231 cells (Fig. 4D, H). These results indicate that PRMT1-mediated meR342-EZH2 is able to repress *P16* and *P21* transcriptional expression. Therefore, we speculated that meR343-EZH2 might lead to H3K27me3 enrichment in *P16* and *P21* promoter regions.

We carried out ChIP assays to verify these hypotheses in MDA-MB-231 cells. First, we showed that silencing PRMT1 expression led to a decrease in EZH2 and H3K27me3 at the *P16* and *P21* promoters (Fig. 4I, J). Moreover, we demonstrated that only overexpression of Flag-EZH2-WT could strongly decrease *P16* and *P21* promoter enrichment of EZH2 and H3K27me3, compared with their counterparts in MDA-MB-231 cells overexpressing Flag-EZH2-R342K (Fig. 4K, L). Our data strongly suggest that meR342-EZH2 suppresses *P16* and *P21* transcription by enhancing H3K27me3 enrichment on these gene promoters.

### meR342-EZH2 promotes breast cancer tumorigenesis in vivo

**Fig. 5:**
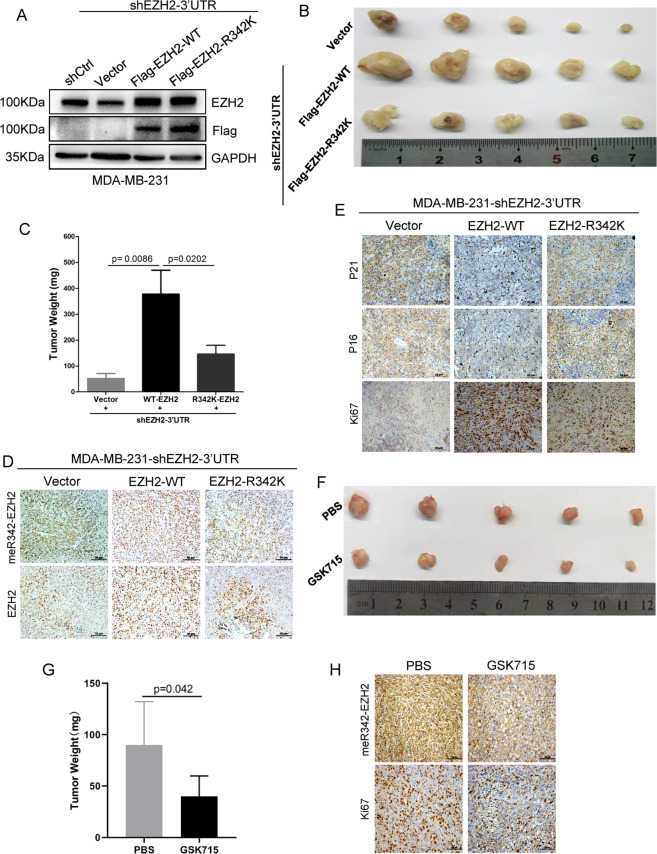
meR342-EZH2 promotes breast cancer tumorigenesis in vivo. **A** Western blot assays confirmed knockdown EZH2 and overexpression of EZH2 in MDA-MB-231 cells. **B**, **C** Here, 5 × 10^6^ MDA-MB-231-Vector/Flag-EZH2-WT/Flag-EZH2-R342K cells were subcutaneously injected into each mice. *n* = 5 for each group. Four weeks after injection, the mice were killed and the xenograft tumours were collected (**B**), and the weight of the xenograft tumours was analysed (**C**). **D**, **E** IHC staining assays were performed in obtained xenograft tumours to detect EZH2, meR342-EZH2, P16, P21 and Ki67. **F**, **G** Here, 5 × 10^6^ MDA-MB-231 cells were subcutaneously injected into each mice. *n* = 5 for each group. Three weeks after injection, the mice were treated with GSK715 (100 mg/kg, each day) or PBS. After 10 days treatment with GSK715 or PBS, the nude mice were killed and the xenograft tumours were collected (**F**), and the weight of the xenograft tumours was analysed (**G**). **H** IHC staining assays were performed in collected xenograft tumours treated with GSK715 or PBS, to detect meR342-EZH2 and Ki67 expression.

We intended to explore whether PRMT1-mediated meR342-EZH2 is required for breast cancer cell proliferation in vivo by a subcutaneous xenograft mouse model assay. MDA-MB-231-shEZH2-3′UTR-vector cells (vector group), MDA-MB-231-shEZH2-3′UTR-Flag-EZH2-WT cells (EZH2-WT group) or MDA-MB-231-shEZH2-3′UTR-Flag-EZH2-R342K cells (EZH2-R342K group) were subcutaneously injected into BALB/c female nude mice. Thirty days later, these mice were killed and mouse-formed tumours were detected. Our results showed that tumours of the EZH2-WT groups were much larger and heavier than those of the vector group or the EZH2-R342K group; nevertheless, the size and weight of tumours in the EZH2-R342K group and the vector group showed little change (Fig. 5A–C). These results indicate that meR342-EZH2 is necessary for breast cancer tumorigenesis in vivo.

Subsequently, we detected the expression of several important markers in tumour tissue sections by IHC assays. We confirmed the high EZH2 expression in the EZH2-WT and EZH2-R342K groups compared with the vector group (Fig. 5D). Then, we also verified that meR342-EZH2 was highly expressed only in the EZH2-WT group (Fig. 5D). Moreover, we found that Ki67 expression in tumours (a cell proliferation marker) was much higher in the EZH2-WT group than in the other two groups (Fig. 5E). Finally, we also measured P16 and P21 expression in the tumour tissues of the three groups formed. The results showed that the expression of P16 and P21 was strongly decreased only in tumours of the EZH2-WT group (Fig. 5E).

Besides, we also detected the PRMT1-specific inhibitor GSK715 functions in tumour growth by the above mentioned xenograft mouse model assay. Our results revealed that GSK715 strongly inhibits breast cancer cells tumour growth ability (Fig. 5F, G). The IHC assays also demonstrated that both meR342-EZH2 and Ki67 expression are significantly suppressed after GSK715 treatment (Fig. 5H). Our data indicate that PRMT1 inhibitor GSK715 can repress breast cancer tumorigenesis, which may mainly through inhibiting PRMT1-mediated meR342-EZH2 methylation. Above all, these results are consistent with our results mentioned in EZH2-WT inhibiting breast cancer cells proliferation through suppressing P16 and P21 expression in vitro. These data indicated that PRMT1-mediated meR342-EZH2 has an important effect on breast cancer tumorigenesis in vivo.

### meR342-EZH2 is negatively correlated with pT311-EZH2 expression in breast cancer patients

**Fig. 6:**
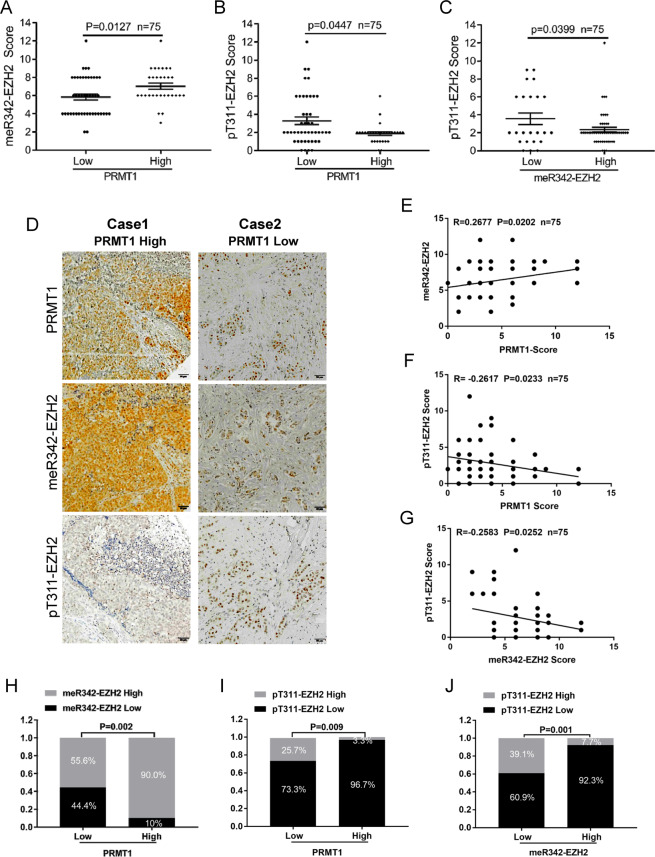
PRMT1-mediated meR342-EZH2 is negatively correlated with pT311-EZH2 expression in breast cancer specimens. **A**–**C** IHC assays in breast cancer tissues were measured using anti-PRMT1, anti-meR342-EZH2 and anti-pT311-EZH2 antibodies (*n* = 75). Semi-quantitative scoring method (using a scale from 0 to 12) was used to quantify the scores of PRMT1, meR342-EZH2 and pT311-EZH2 IHC staining. Analysing the relevant meR342-EZH2 (**A**) or pT311-EZH2 (**B**) expression in PRMT1-low cases and PRMT1-high cases; analysing the relevant pT311-EZH2 expression in meR342-EZH2-low cases and meR342-EZH2-high cases (**C**) by Student’s *t*-test. **D** Representative images of PRMT1, meR342-EZH2 and pT311-EZH2 expressions in PRMT1-high case and PRMT1-low case were presented. **E**–**G** Correlation between PRMT1 and meR342-EZH2 expression (**E**), PRMT1 and pT311-EZH2 expression (**F**), meR342-EZH2 and pT311-EZH2 (**G**) were examined by Pearson’s correlation coefficient test, respectively (*n* = 75). **H**–**J** Correlation between PRMT1 and meR342-EZH2 expression (**H**), PRMT1 and pT311-EZH2 expression (**I**), meR342-EZH2 and pT311-EZH2 (**J**) were examined by Fisher’s exact test, respectively (*n* = 75).

Finally, to evaluate the clinical significance of our findings, we conducted IHC assays in breast cancer TMA slides using anti-PRMT1, anti-meR342-EZH2 and anti-pT311-EZH2 antibodies. The data showed that meR342-EZH2 was highly expressed in breast cancer patients with high PRMT1 expression (Fig. 6A), whereas pT311-EZH2 was expressed at low levels in breast cancer tissues with high PRMT1 expression (Fig. 6B). Moreover, we also discovered that pT311-EZH2 expression was low when meR342-EZH2 was highly expressed in breast cancer tissues (Fig. 6C). We also analysed the expression correlation among PRMT1, meR342-EZH2 and pT311-EZH2 (Fig. 6D–G). We found that PRMT1 was positively correlated with meR342-EZH2 expression (Fig. 6E). At the same time, our results also showed that both PRMT1-pT311-EZH2 expression and meR342-EZH2-pT311-EZH2 expression had a negative correlation (Fig. 6F, G). In addition, we observed that high PRMT1 expression positively correlated with high meR342-EZH2 expression but negatively correlated with high pT311-EZH2 expression (Fig. 6H, I). Furthermore, we showed that high meR342-R342 positively correlated with low pT311-EZH2 expression in breast cancer tissues (Fig. 6J). Overall, these clinical data indicated that PRMT1 and meR342-EZH2 expression has a positive correlation in breast cancer patients. In contrast, meR342-EZH2 and pT311-EZH2 expression had a negative correlation in the detected breast cancer tissues.

## Discussion

In this study, we demonstrated that PRMT1 catalyses EZH2-R342 methylation and is necessary for PRMT1 to promote cell proliferation in breast cancer in vitro and in vivo. We clarified that meR342-EZH2 strengthens the EZH2-SUZ12 interaction and subsequent PRC2 aggregation by disrupting AMPKα1 phosphorylation of EZH2 at the T311 site. We also verified the PRMT1-meR342-EZH2-positive expression correlation and meR342-EZH2-pT311-EZH2 -negative expression correlation in breast cancer tissue samples.

Recently, a series of studies have discovered that PRMT1 can catalyse varies substrates methylation. In addition, some of these substrate methylation modifications have effect on cancer cells proliferation. For instance, reports reveal that PRMT1-mediated BRCA1 and c-Myc methylations can regulate cancer cells cell cycle progression and proliferation [32, 33]. Interestingly, we showed that PRMT1 also can enhance breast cancer cell proliferation after inhibiting BRCA1 or c-Myc functions by their specific inhibitor (Supplementary Fig. S4). These findings in this study strongly suggest that PRMT1-mediated meR342-EZH2 is required for breast cancer cells proliferation. Further, PRMT1-mediated EZH2 methylation may play a critical part in PRMT1-induced cell proliferation acceleration.

A series of studies reported that protein arginine methylation is able to engage in crosstalk with proteins that undergo phosphorylation modification. For example, a previous study demonstrated that PRMT5-mediated EGFR-R1175 methylation facilitates epidermal growth factor-induced epidermal growth factor receptor (EGFR) phosphorylation at Y1173, which inhibits EGFR-mediated ERK activation [34]. Here we revealed that PRMT1-mediated meR342-EZH2 disrupts AMPK phosphorylation at EZH2-T311. Recently, we revealed that arginine methylation is able to regulate protein spatial structure [28]. Therefore, we proposed that PRMT1-mediated meR342-EZH2 probably alters the EZH2 spatial structure, which prevents AMPK binding with and phosphorylating EZH2.

Several PRMT1-specific inhibitors have been developed. It has been reported that many PRMT1 inhibitors can attenuate cancer cell growth in vitro and in vivo [1, 35]. It has been reported that the PRMT1 inhibitor GSK715 is in a phase I clinical trial of cancer treatment [35]. In this study, we observed that the PRMT1 inhibitors AMI-1 and GSK715 significantly inhibited MDA-MB-231 breast cancer cell proliferation. Therefore, we speculate that the PRMI1 inhibitor GSK715 may become a promising anticancer drug for targeting PRMT1. Moreover, a previous study confirmed that metformin can disrupt breast cancer cell proliferation by stimulating AMPK-mediated pT311-EZH2 [26]. We believe that a PRMT1-specific inhibitor in combination with metformin will be a promising therapeutic strategy to prevent breast cancer progression in patients.

## Supplementary information

Supplementary Information

Supplementary Figure Legends

Supplementary Figure 1

Supplementary Figure 2

Supplementary Figure 3

Supplementary Figure 4

Related Manuscript File

## Data Availability

The data used and/or analysed during the current study are available from the corresponding author on reasonable request.
