# Supplementary material for: PRMT1-mediated EZH2 methylation promotes breast cancer cell proliferation and tumorigenesis
Source: Cell Death Dis. 2021 Nov 13;12(11):1080. doi: 10.1038/s41419-021-04381-5 (PMC8590688; doi:10.1038/s41419-021-04381-5)
Supplement: Supplementary file 1 — Supplementary Information [file 41419_2021_4381_MOESM1_ESM.docx]

**Supplementary Information for**

**PRMT1-mediated EZH2 methylation promotes breast cancer cell proliferation and tumorigenesis**

Zhongwei Li^1,2,#^, Diandian Wang^1,3,#^, Xintian Chen^1,#^, Wenwen Wang^1,#^, Pengfei Wang^1^, Pingfu Hou^1,2^, Minle Li^1,2^, Sufang Chu^1^, Shuxi Qiao^1^, Junnian Zheng^1,2,*^ and Jin Bai^1,2,*^

**Affiliations**

^1^Cancer Institute, Xuzhou Medical University, Xuzhou, Jiangsu, China.

^2^Center of Clinical Oncology, Affiliated Hospital of Xuzhou Medical University, Xuzhou, Jiangsu, China.

^3^Intensive Care Unit, The Second Affiliated Hospital of Xuzhou Medical University, Xuzhou, Jiangsu, China.

^#^These authors contributed equally to this paper.

^*^Correspondence authors: Jin Bai, E-mail: bj@xzhmu.edu.cn ; Junnian Zheng, E-mail: jnzheng@xzhmu.edu.cn.

**1 shRNA, qRT-PCR primer and ChIP assay primer sequences**

shCtrl targeting sequence: TTCTCCGAACGTGTCACGT

shEZH2-3’UTR targeting sequence: GCTGCCTTAGCTTCAGGAA

shPRMT1#1 targeting sequence: GCGAGGAGATCTTCGGCACCA

shPRMT1#2 targeting sequence: GGACATGACATCCAAAGAT

GAPDH qRT-PCR Forward primer: ATGACCCCTTCATTGACCTCA

GAPDH qRT-PCR Reverse primer: GAGATGATGACCCTTTTGGCT

P16 qRT-PCR Forward primer: CATAGATGCCGCGGAAGGT

P16 qRT-PCR Reverse primer: CCCGAGGTTTCTCAGAGCCT

P21 qRT-PCR Forward primer: TTTCTCTCGGCTCCCCATGT

P21 qRT-PCR Reverse primer: GCTGTATATTCAGCATTGTGGG

P16 ChIP Forward primer: CCCCGATTCAATTTGGCAGTTAGG

P16 ChIP Reverse primer: CAGCGTTGGCAAGGAAGGAGGAC

P21 ChIP Forward primer: GGTGTCTAGGTGCTCCAGGT

P21 ChIP Reverse primer: GCACTCTCCAGGAGGACACA

**2 Reagents**

| **REAGENT or RESOURCE** | **SOURCE** | **IDENTIFIER** |
| --- | --- | --- |
| AMI-1 | Selleckchem | Cat#S7884 |
| Bractoppin | MedChemExpress | Cat#HY-126020 |
| GSK3368715 dihydrochloride  (short for GSK715) | MedChemExpress | Cat# HY-128717A |
| 10074-G5 | MedChemExpress | Cat#HY-100996 |
| DMEM | Sigma-Aldrich | Cat#D7777 |
| RPMI-1640 | Sigma-Aldrich | Cat#R7755 |
| DMEM/F12 | Sigma-Aldrich | Cat#D8900 |
| Fetal Bovine Serum | TRANSGEN BIOTECH | Cat#FS201-02 |
| L15 | Sigma-Aldrich | Cat#L4386 |

**3 Cell cycle analysis**

Forty-eight hours after transfection, the cells were synchronized by serum starvation overnight and induced re-enter cell cycle by incubating in medium containing 10% fetal bovine serum for 4 h. Then the cells were collected, washed by PBS, and fixed in pro-cooled 70% ethanol at 4 °C overnight. The day after, the cells were washed twice with PBS and resuspended in RNase A at 37 °C for 30 min, and then propidium iodide (PI) was added to the cells in the dark at 4 °C for 30 min. In the end, all samples were analyzed by flow cytometry (BD, FACSCantoTM II).

**4 Method of IHC assessment**

PRMT1, meR342-EZH2 and EZH2 staining were evaluated blindly and independently by different pathologists. The signals were quantified according to both the intensity and percentage of cells with positive staining. The PRMT1, meR342-EZH2 and EZH2 staining intensity was scored 0 to 3 (0 = negative; 1 = weak; 2 = moderate; 3 = strong). The percentage of positive stained cells was also scored into four categories: 1 (0%-25%), 2 (26%-50%), 3 (51%-75%) and 4 (76%-100%). The IHC Score level of PRMT1, meR342-EZH2 and EZH2 staining was evaluated by IRS, which is calculated by multiplying the scores of staining intensity and the percentage of positive cells. Based on IRS, the PRMT1, meR342-EZH2 and EZH2 staining pattern was categorized as negative (IRS: 0), weak (IRS: 1-3), moderate (IRS: 4-6) and strong (IRS: 8-12).
