# Supplementary material for: PRMT1-mediated EZH2 methylation promotes breast cancer cell proliferation and tumorigenesis
Source: Cell Death Dis. 2021 Nov 13;12(11):1080. doi: 10.1038/s41419-021-04381-5 (PMC8590688; doi:10.1038/s41419-021-04381-5)
Supplement: Supplementary file 2 — Supplementary Figure Legends [file 41419_2021_4381_MOESM2_ESM.docx]

**Supplementary Figure Legends**

**Supplementary Figure 1.** CCK-8 assays measured cell proliferation ability after treated with GSK715 (0.31 nM) in MCF7 cells and MDA-MB-231 cells, respectively.

**Supplementary Figure 2.** Western blot detecting EZH2 binding with meR342-EZH2, AMPKα1, SUZ12, EED after Co-IP Flag-EZH2 in HEK293T-Flag-EZH2-WT and HEK293T-Flag-EZH2-R342K cells.

**Supplementary Figure 3.** Western blot detection of P16 and P21 expression after knockdown PRMT1 in MCF7 cell.

**Supplementary Figure 4. A** CCK-8 detected the MCF7-Vector cells and MCF7-PRMT1 cells proliferation ability after treated with BRCA1 inhibitor Bractoppin (74 nM), respectively. B CCK-8 detected the MCF7-Vector cells and MCF7-PRMT1 cells proliferation ability after treated with c-Myc inhibitor 10074-G5 (146 μM).
