# Supplementary figures and images for: PRMT1-mediated EZH2 methylation promotes breast cancer cell proliferation and tumorigenesis
Source: Cell Death Dis. 2021 Nov 13;12(11):1080. doi: 10.1038/s41419-021-04381-5 (PMC8590688; doi:10.1038/s41419-021-04381-5)

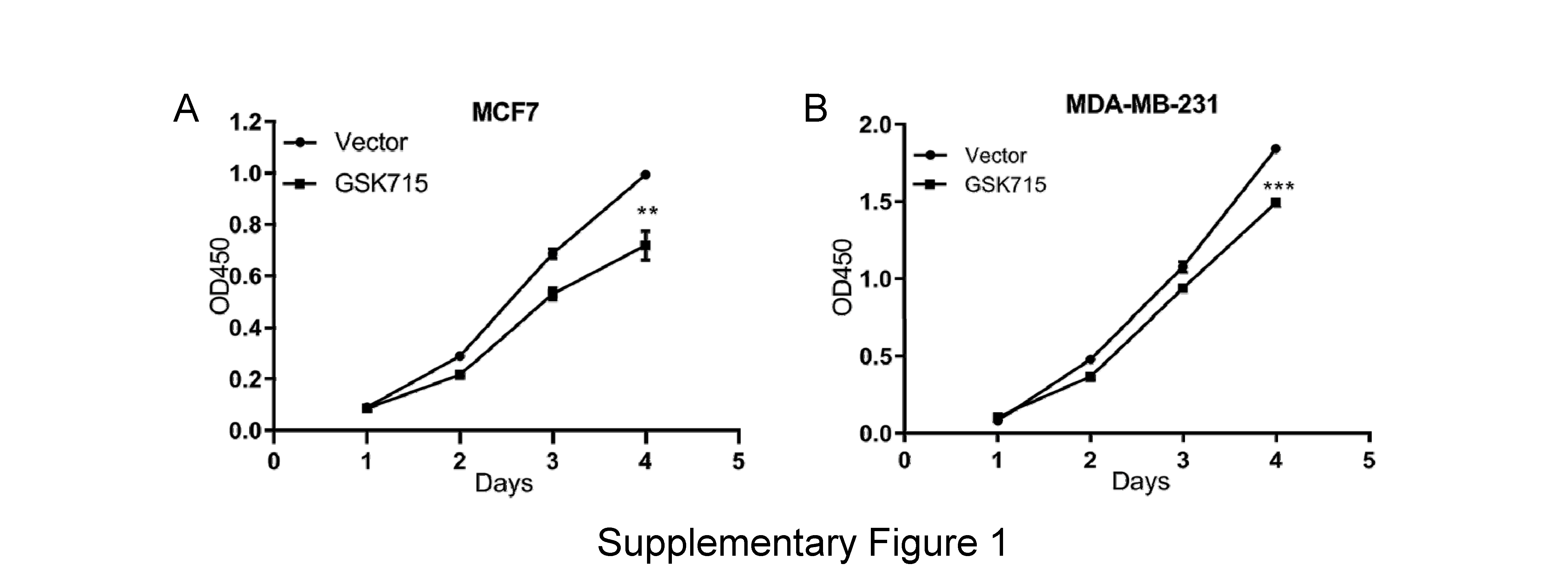

Supplement: Supplementary file 3 — Supplementary Figure 1 [file 41419_2021_4381_MOESM3_ESM.png]

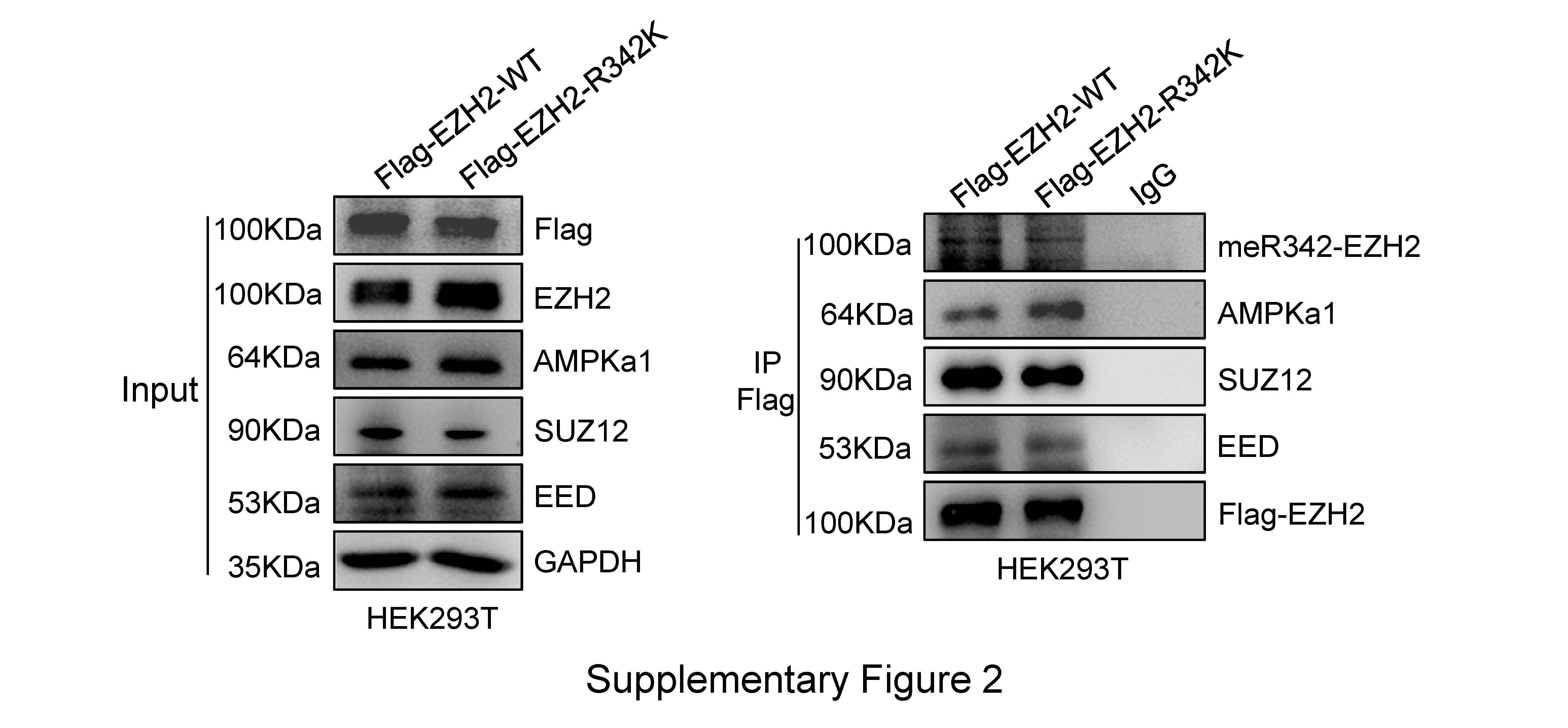

Supplement: Supplementary file 4 — Supplementary Figure 2 [file 41419_2021_4381_MOESM4_ESM.png]

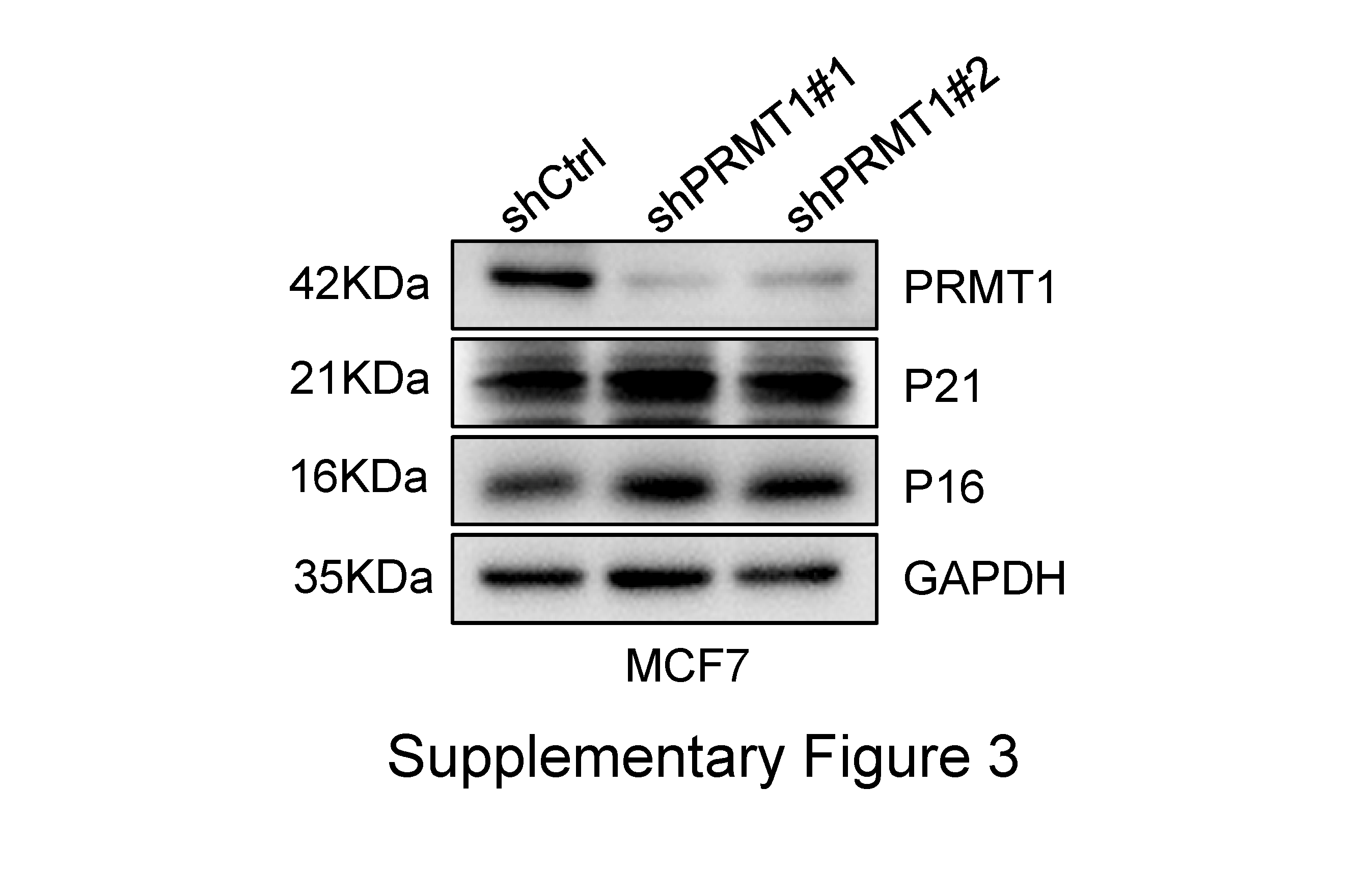

Supplement: Supplementary file 5 — Supplementary Figure 3 [file 41419_2021_4381_MOESM5_ESM.png]

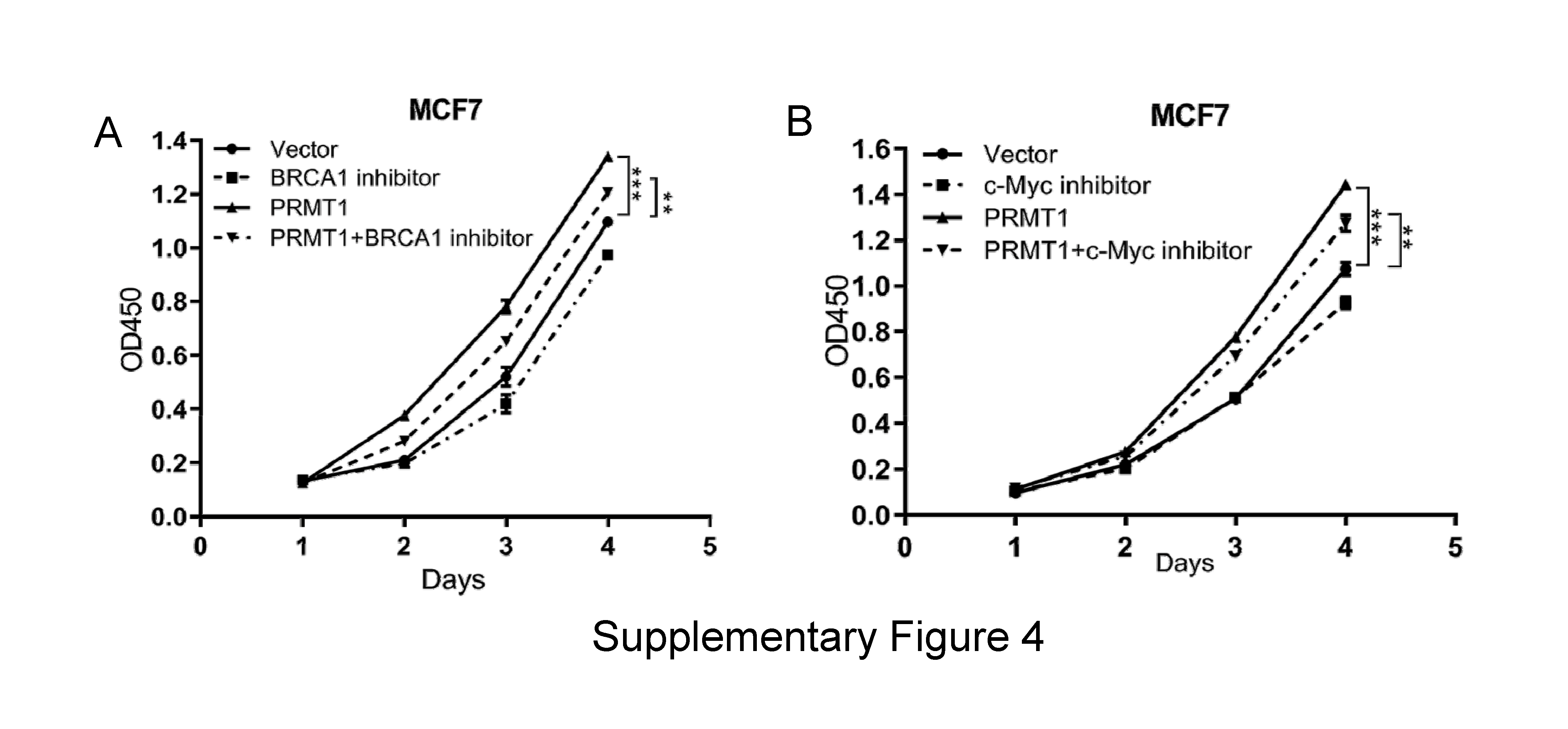

Supplement: Supplementary file 6 — Supplementary Figure 4 [file 41419_2021_4381_MOESM6_ESM.png]
