# Supplementary material for: PRMT1-mediated EZH2 methylation promotes breast cancer cell proliferation and tumorigenesis
Source: Cell Death Dis. 2021 Nov 13;12(11):1080. doi: 10.1038/s41419-021-04381-5 (PMC8590688; doi:10.1038/s41419-021-04381-5)
Supplement: Supplementary file 7 — Related Manuscript File [file 41419_2021_4381_MOESM7_ESM.pdf]

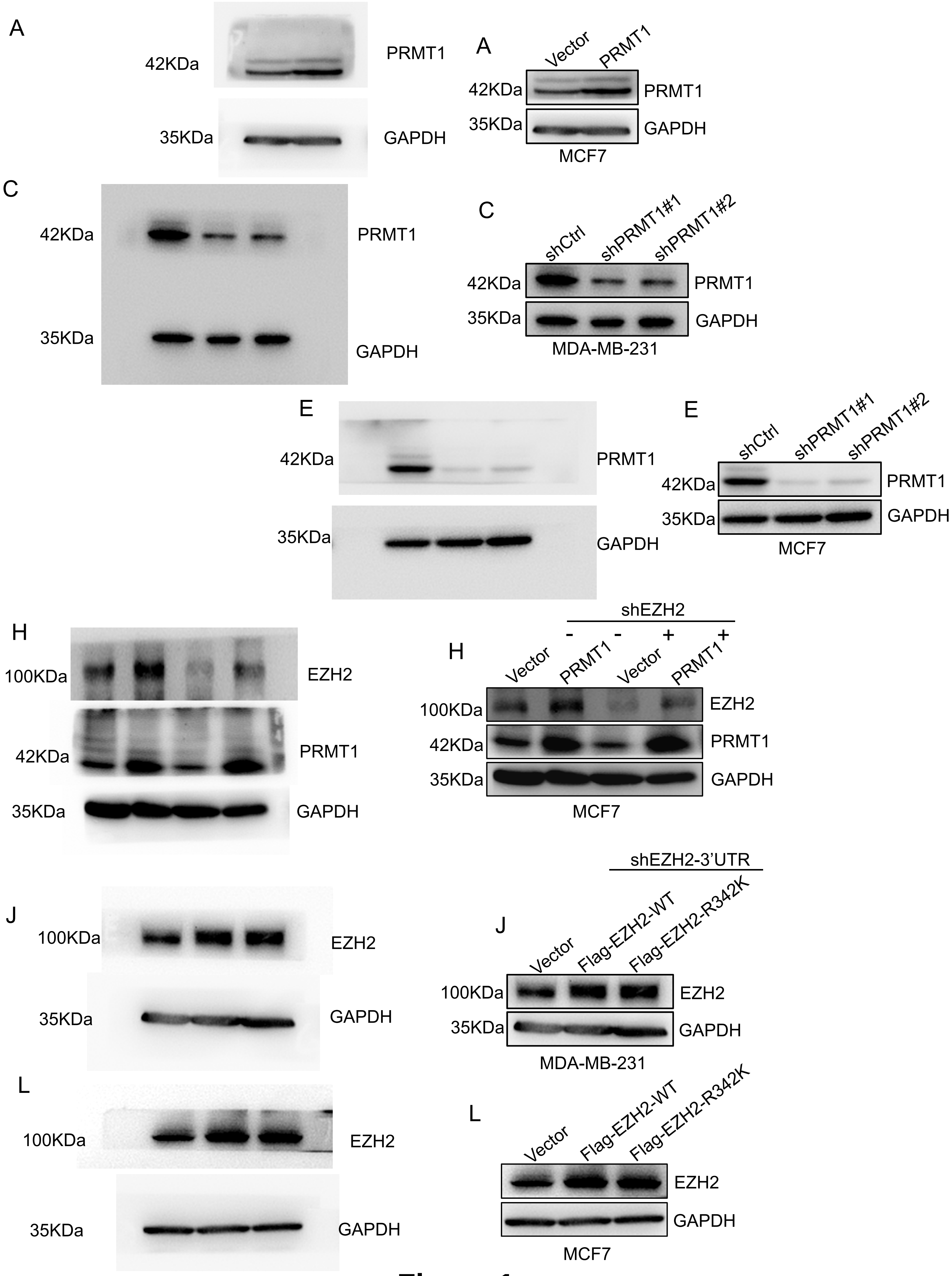
A
42KDa
PRMT1
35KDa
GAPDH
A
Vector
PRMT1
42KDa
PRMT1
35KDa
GAPDH
MCF7
C
42KDa
PRMT1
35KDa
GAPDH
C
shCtrl
shPRMT1#1
shPRMT1#2
42KDa
PRMT1
35KDa
GAPDH
MDA-MB-231
E
42KDa
PRMT1
35KDa
GAPDH
E
shCtrl
shPRMT1#1
shPRMT1#2
42KDa
PRMT1
35KDa
GAPDH
MCF7
H
100KDa
EZH2
42KDa
PRMT1
35KDa
GAPDH
shEZH2
- - + +
H
Vector
PRMT1
Vector
PRMT1
100KDa
EZH2
42KDa
PRMT1
35KDa
GAPDH
MCF7
J
100KDa
EZH2
35KDa
GAPDH
shEZH2-3'UTR
J
Vector
Flag-EZH2-WT
Flag-EZH2-R342K
100KDa
EZH2
35KDa
GAPDH
MDA-MB-231
L
100KDa
EZH2
35KDa
GAPDH
L
Vector
Flag-EZH2-WT
Flag-EZH2-R342K
EZH2
GAPDH
MCF7

Figure 1

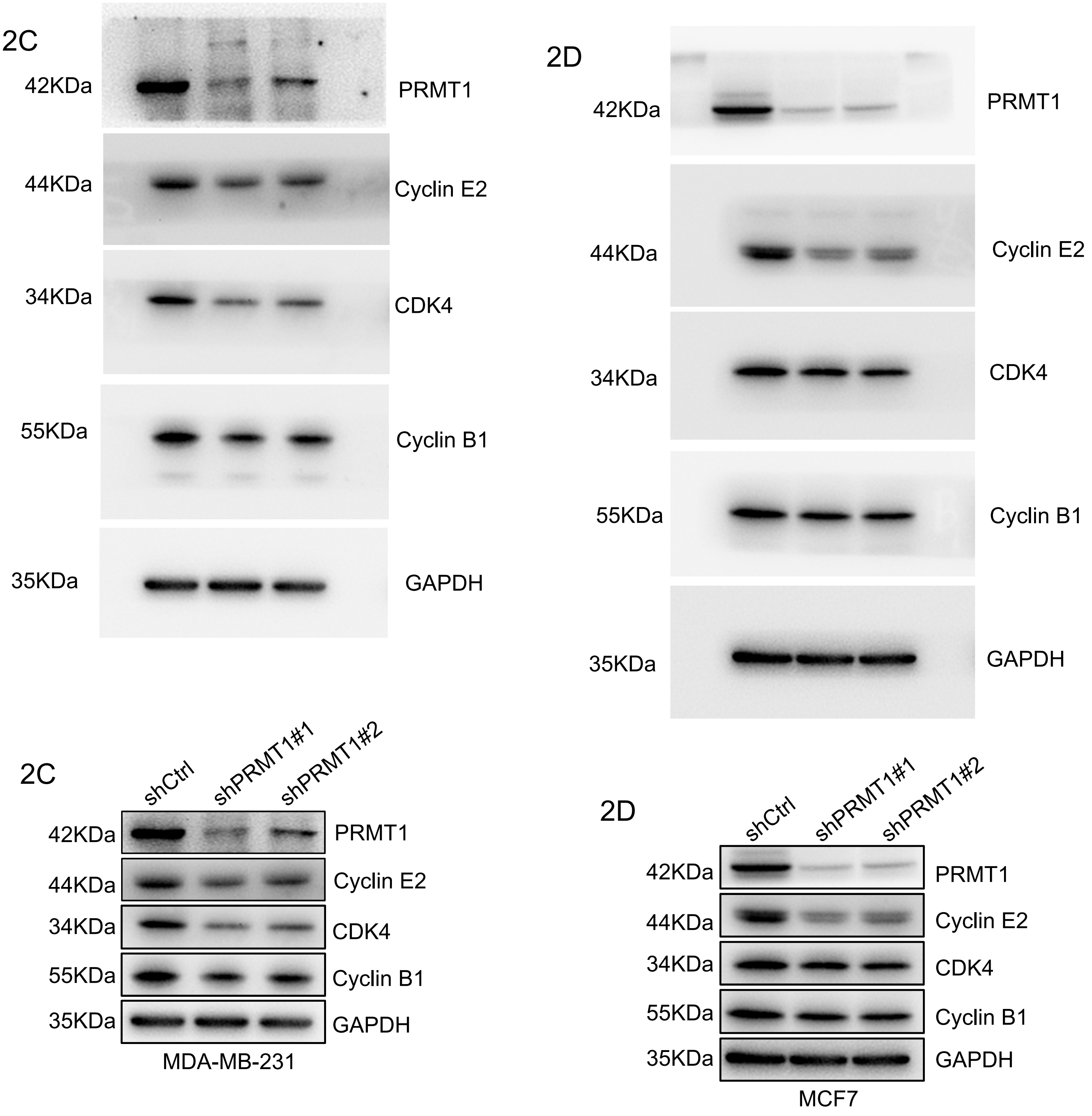
2C
42KDa
PRMT1
44KDa
Cyclin E2
34KDa
CDK4
55KDa
Cyclin B1
35KDa
GAPDH
2D
42KDa
PRMT1
44KDa
Cyclin E2
34KDa
CDK4
55KDa
Cyclin B1
35KDa
GAPDH
2C
shCtrl
shPRMT1#1
shPRMT1#2
42KDa
PRMT1
44KDa
Cyclin E2
34KDa
CDK4
55KDa
Cyclin B1
35KDa
GAPDH
MDA-MB-231
2D
shCtrl
shPRMT1#1
shPRMT1#2
42KDa
PRMT1
44KDa
Cyclin E2
34KDa
CDK4
55KDa
Cyclin B1
35KDa
GAPDH
MCF7

Figure 2

2E

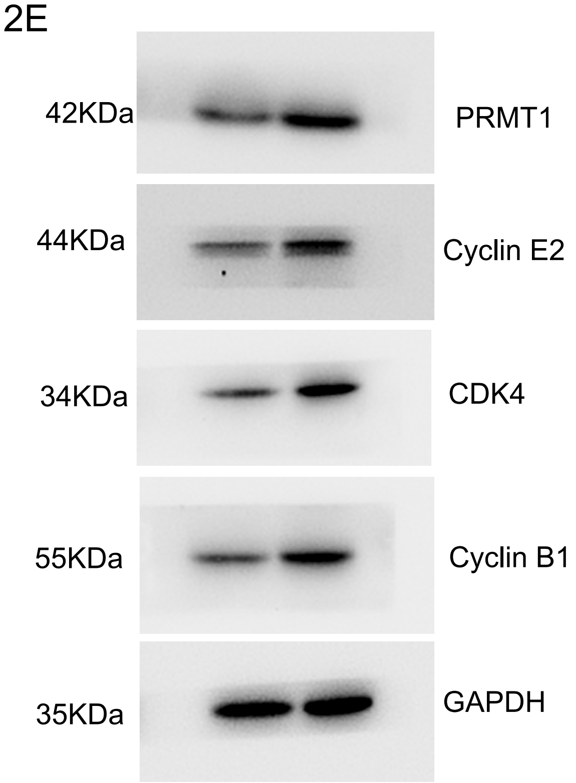

2F

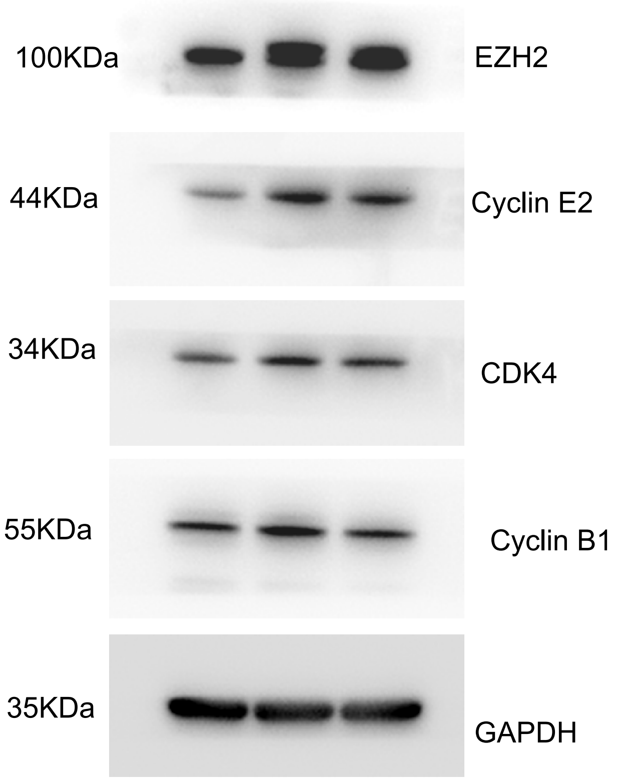

2E

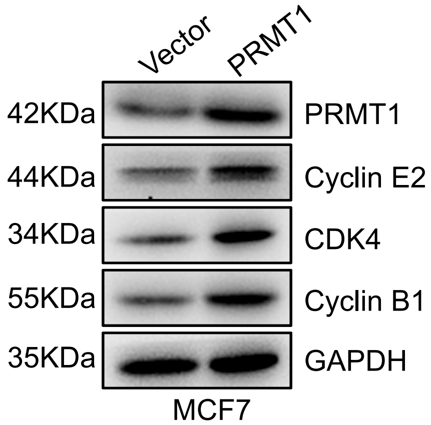

2F

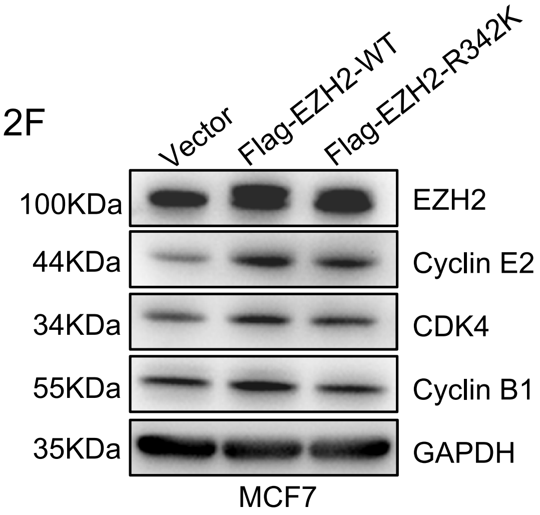

Figure 2----continue

3A

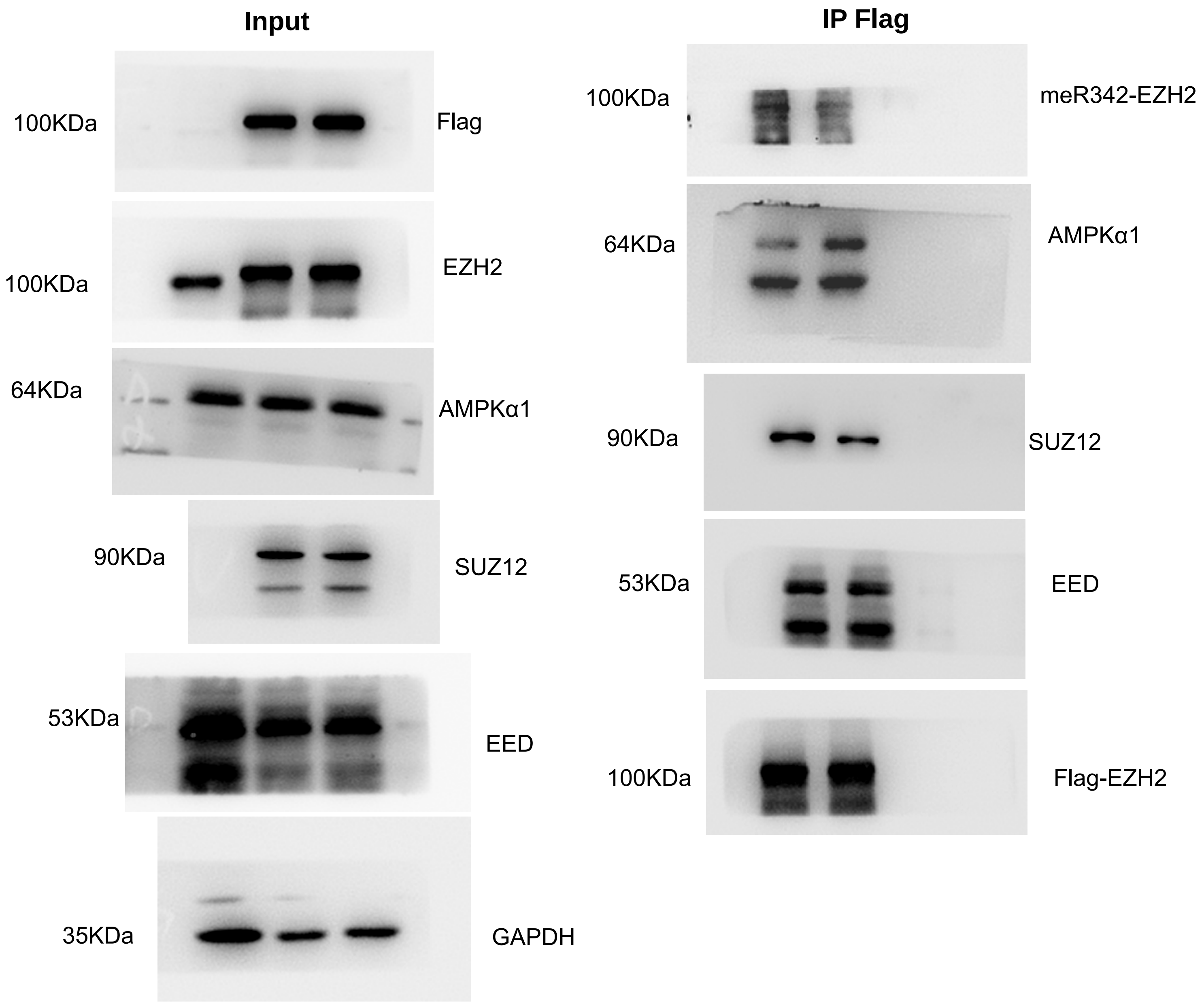

3A

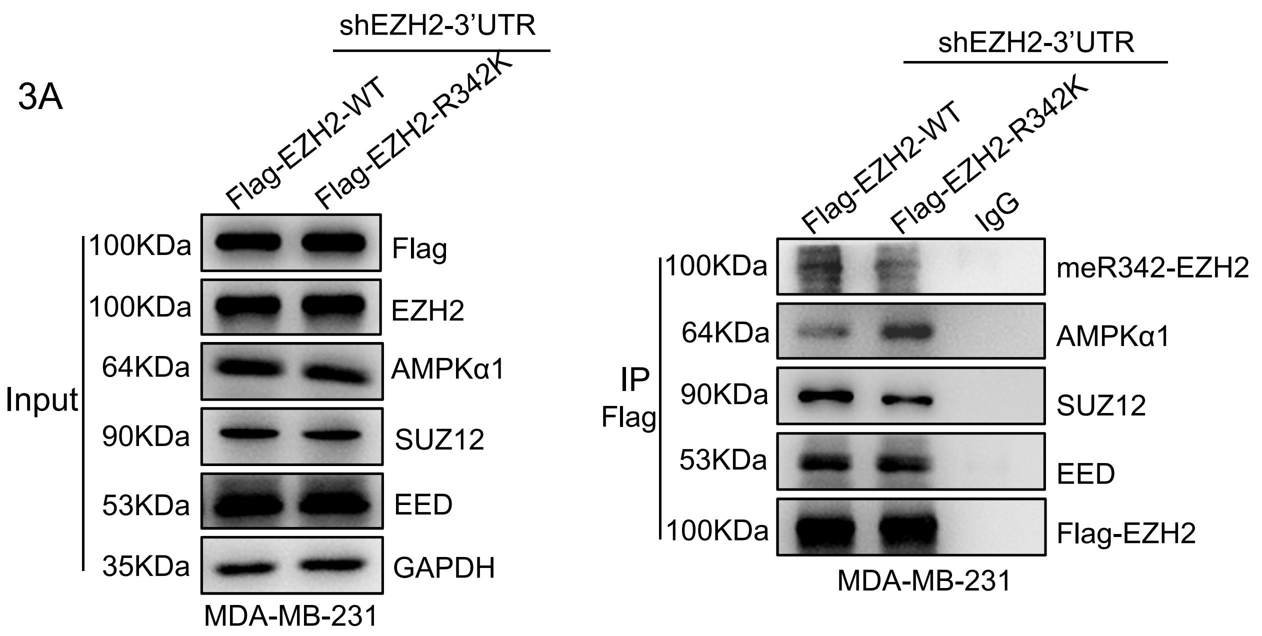

Figure 3

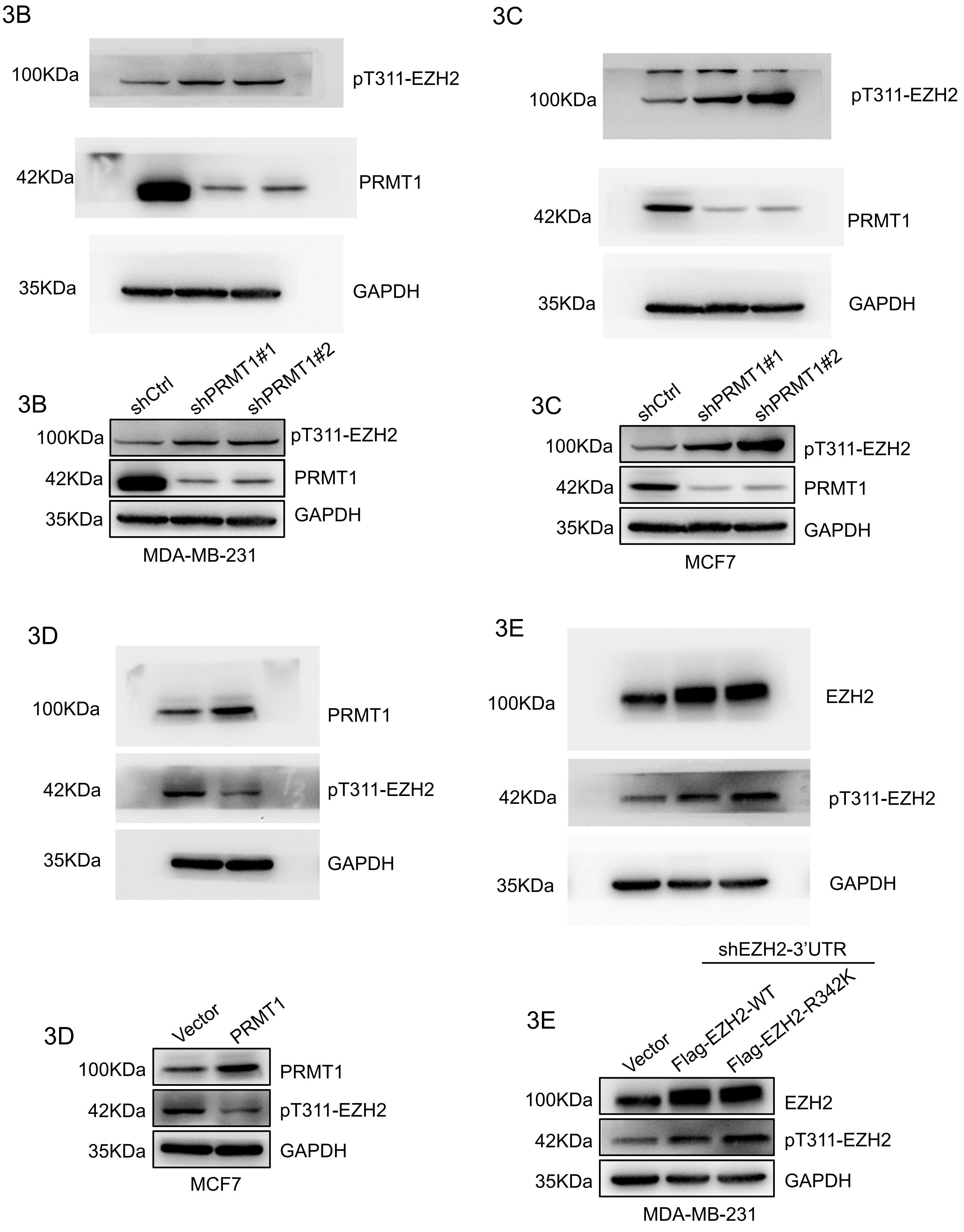

Figure 3---continue

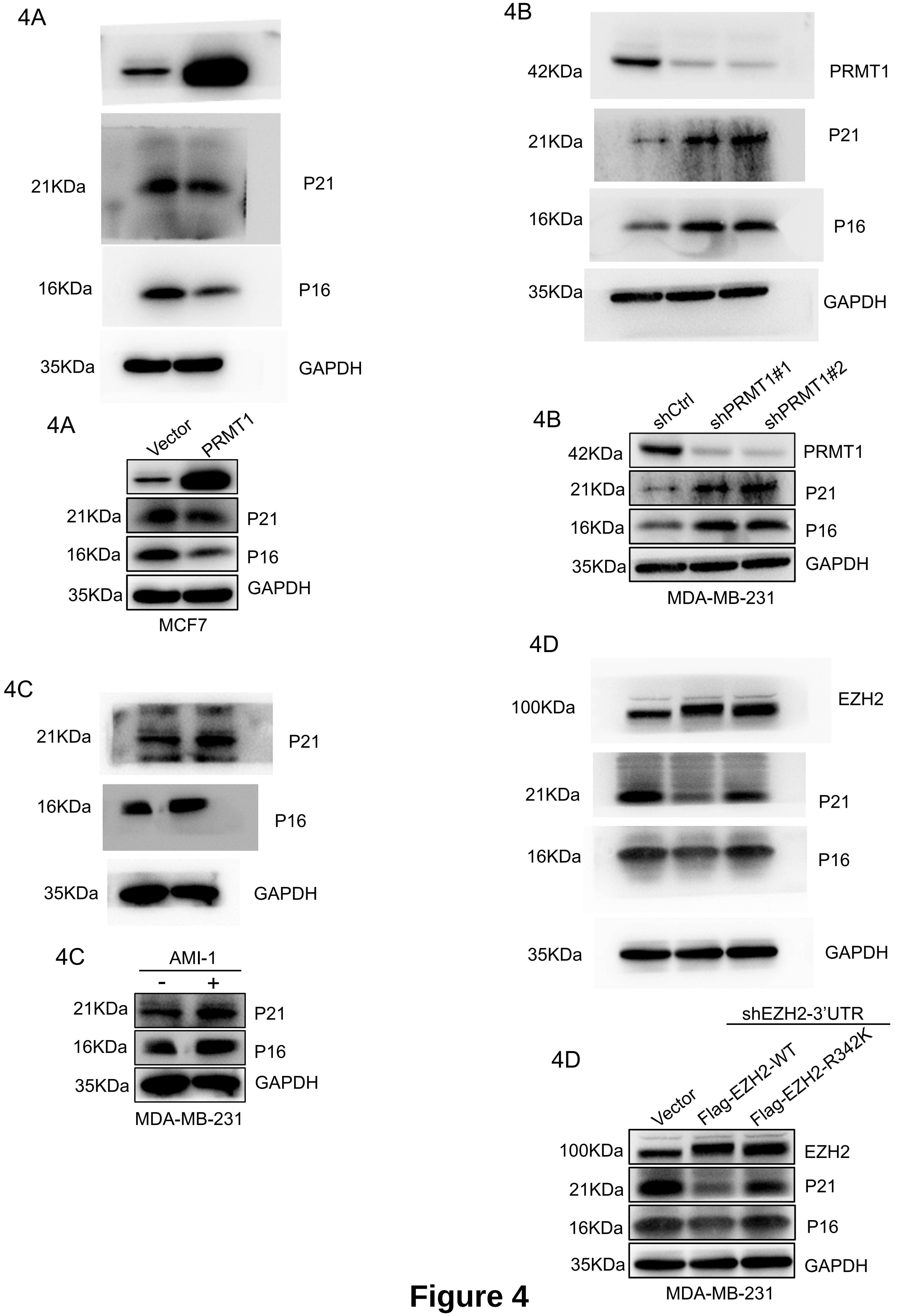
4A
21KDa
P21
16KDa
P16
35KDa
GAPDH
4A
Vector
PRMT1
21KDa
P21
16KDa
P16
35KDa
GAPDH
MCF7
4B
42KDa
PRMT1
21KDa
P21
16KDa
P16
35KDa
GAPDH
4B
shCtrl
shPRMT1#1
shPRMT1#2
42KDa
PRMT1
21KDa
P21
16KDa
P16
35KDa
GAPDH
MDA-MB-231
4C
21KDa
P21
16KDa
P16
35KDa
GAPDH
4C
AMI-1
-
+
21KDa
P21
16KDa
P16
35KDa
GAPDH
MDA-MB-231
4D
100KDa
EZH2
21KDa
P21
16KDa
P16
35KDa
GAPDH
shEZH2-3'UTR
4D
Vector
Flag-EZH2-WT
Flag-EZH2-R342K
100KDa
EZH2
21KDa
P21
16KDa
P16
35KDa
GAPDH
MDA-MB-231

Figure 4

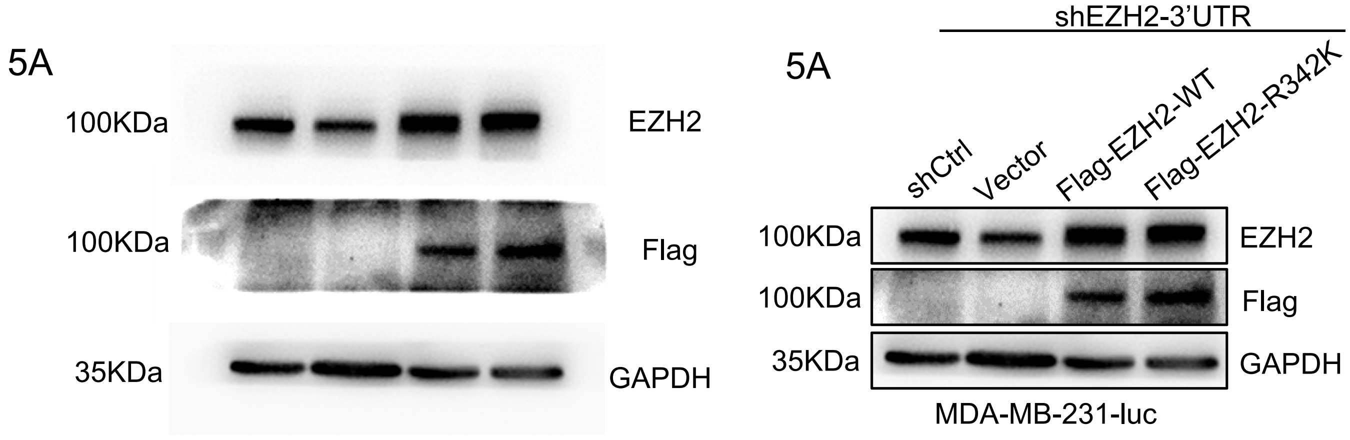
5A
100KDa
EZH2
100KDa
Flag
35KDa
GAPDH
shEZH2-3'UTR
5A
shCtrl
Vector
Flag-EZH2-WT
Flag-EZH2-R342K
100KDa
EZH2
100KDa
Flag
35KDa
GAPDH
MDA-MB-231-luc

Figure 5

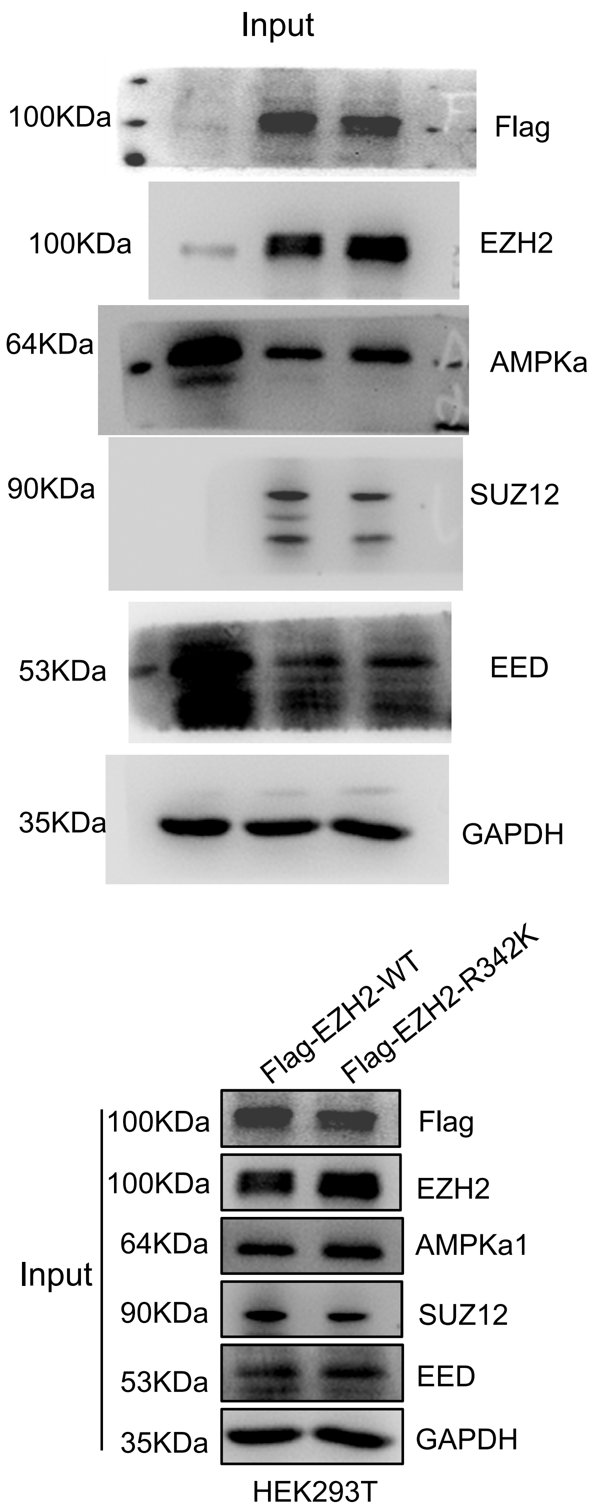

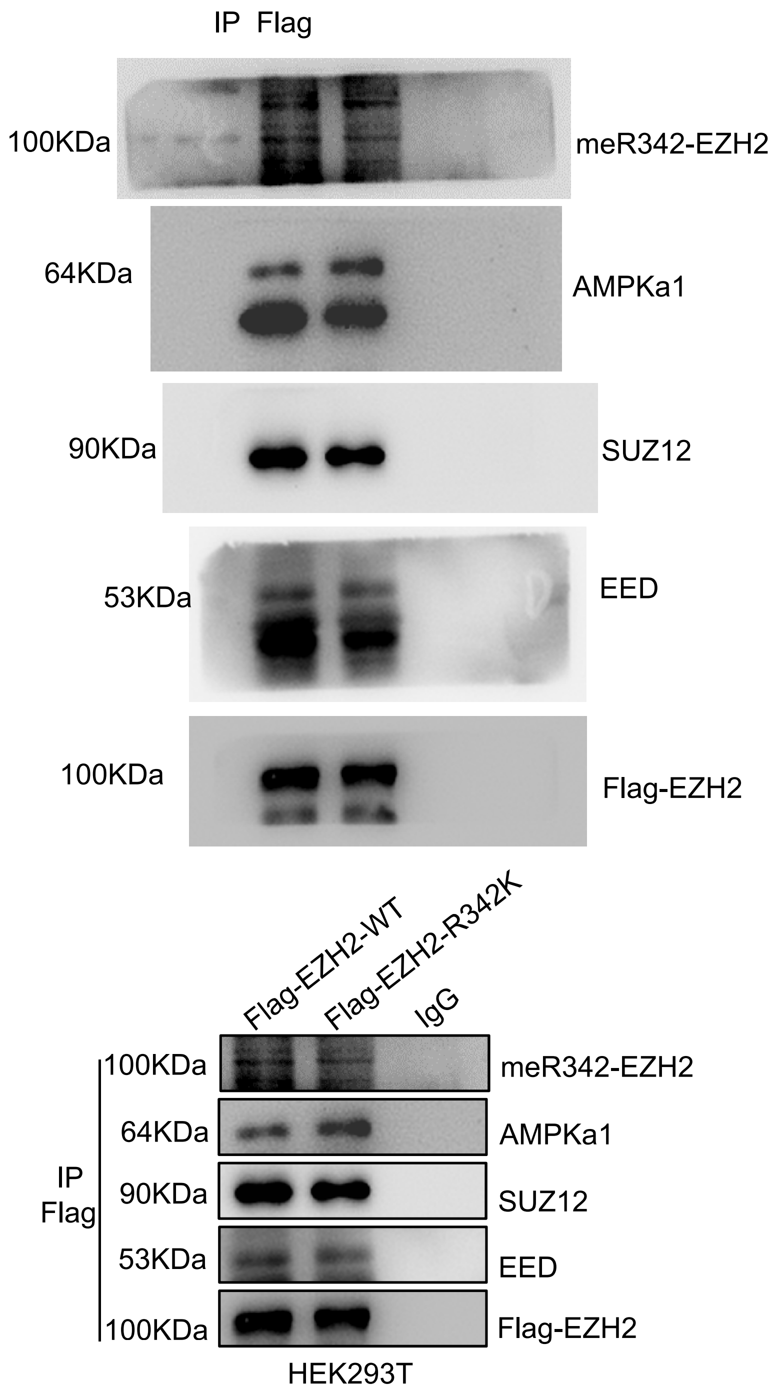

Supplementary Figure 2

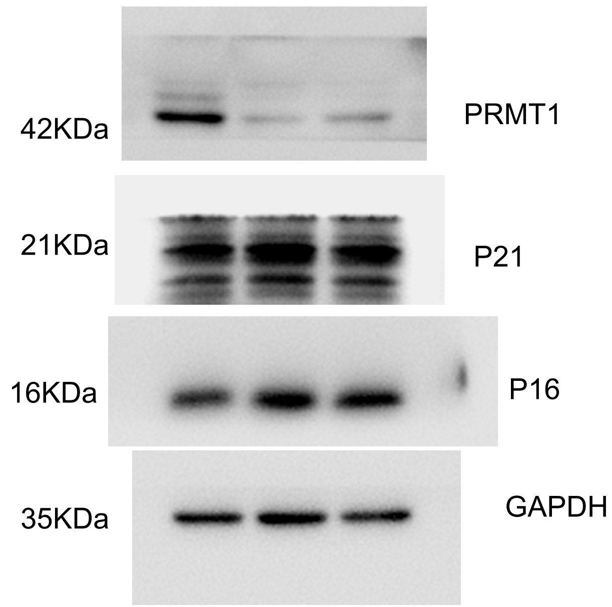

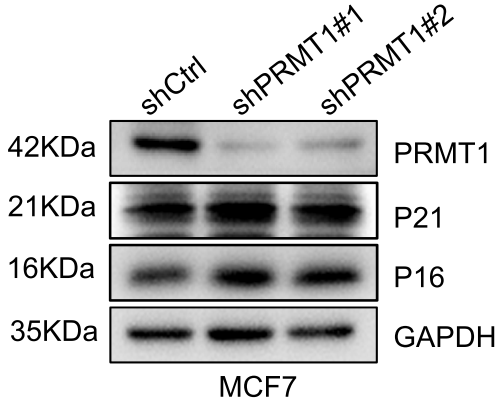

Supplementary Figure 3
